# High capacity silicon anodes enabled by MXene viscous aqueous ink

**DOI:** 10.1038/s41467-019-08383-y

**Published:** 2019-02-20

**Authors:** Chuanfang (John) Zhang, Sang-Hoon Park, Andrés Seral‐Ascaso, Sebastian Barwich, Niall McEvoy, Conor S. Boland, Jonathan N. Coleman, Yury Gogotsi, Valeria Nicolosi

**Affiliations:** 1 CRANN and AMBER Research Centers, Trinity College Dublin, Dublin 2, Ireland; 2 School of Chemistry, Trinity College Dublin, Dublin 2, Ireland; 3 School of Physics, Trinity College Dublin, Dublin 2, Ireland; 4 A. J. Drexel Nanomaterials Institute and Department of Materials Science and Engineering, Drexel University, Philadelphia, PA 19104 USA; 5 Present Address: School of Mathematical and Physical Sciences, University of Sussex, Sussex, BN1 9QH UK

## Abstract

The ever-increasing demands for advanced lithium-ion batteries have greatly stimulated the quest for robust electrodes with a high areal capacity. Producing thick electrodes from a high-performance active material would maximize this parameter. However, above a critical thickness, solution-processed films typically encounter electrical/mechanical problems, limiting the achievable areal capacity and rate performance as a result. Herein, we show that two-dimensional titanium carbide or carbonitride nanosheets, known as MXenes, can be used as a conductive binder for silicon electrodes produced by a simple and scalable slurry-casting technique without the need of any other additives. The nanosheets form a continuous metallic network, enable fast charge transport and provide good mechanical reinforcement for the thick electrode (up to 450 µm). Consequently, very high areal capacity anodes (up to 23.3 mAh cm^−2^) have been demonstrated.

## Abstract (web summary)

Developing thick electrodes could enable high-energy-density Li-ion batteries, however, above a critical thickness, the mass transport issues become dominating. Here the authors show that MXene can serve as a conductive binder leading to thick silicon anodes (up to 450 µm) with high areal capacity.

## Introduction

Utilization of Li-ion chemistry to store the energy electrochemically can address the ever-increasing demands from both portable electronics and hybrid electrical vehicles^1–4^. Such stringent challenges on the battery safety and lifetime issues require high-performance battery components, with most of the focus being on electrodes or electrolytes with novel nanostructures and chemistries^5–10^. However, equally important is the development of electrode additives, which are required to maintain the electrode’s conductive network and mechanical integrity.

Traditionally, electrode additives are made of dual components based on a conductive agent (i.e. carbon black, CB) and a polymeric binder^11,12^. While the former ensures the charge transport throughout the electrode, the latter mechanically holds the active materials and CB together during cycling. Although these traditional electrode additives have been widely applied in Li-ion battery technologies^13^, they fail to perform well in high-capacity electrodes, especially those displaying large volume changes^14^. This is because the polymeric binder is not mechanically robust enough to withstand the stress induced during lithiation/delithiation, leading to severe disruption of the conducting networks. This results in rapid capacity fade and poor lifetime.

This issue can be solved by employing a conductive binder to accommodate the large volume change of the electrodes^15^. This strategy not only ensures good mechanical adhesion of the active materials to the conductive agent but also decreases the inactive volume/mass of the electrode, leading to improved battery performance. Poly(3,4-ethylenedioxythiophene):poly(styrenesulfonic acid) (PEDOT:PSS)^15^, polyaniline^16^ and polypyrrole^17^ etc.^18,19^ have demonstrated inherent electronic/ionic conductivity in the Li^+^-containing electrolyte and capability in preserving the mechanical integrity of large-capacity materials, such as silicon (Si, *C*_SP_ = ~3500 mAh g^−1^) anodes. However, while several novel conductive binders have been reported for Si-based anodes^20,21^, their achievable areal capacity, which should be more emphasized in practical cases, is generally low (<4 mAh cm^−2^)^22,23^. Revisiting the electrode areal capacity (*C*/*A* = *C*/*M* × *M*/*A*, where *C*/*M* is the specific capacity (mAh g^−1^) of the electrode and *M*/*A* is its mass loading (mg cm^−2^)) implies that one has to increase the *M*/*A* of the Si-based anodes (while retaining high specific capacity). This, in turn, requires the formation of a thick electrode using the commercially available technology to cast the conductive-binder/Si-based slurry. Unfortunately, this has proven to be quite difficult, as the critical cracking thickness (CCT), determined by the viscosity and surface tension of the slurry, etc.^24^, greatly limits the electrodes’ achievable *M*/*A*. This is especially true in the reported conductive polymer solutions, where either concentration (or viscosity) of the solution is fairly low or the capillary pressure in the slurry drying process is too high^14,15^. This means that developing an aqueous solution of conductive-binder with a high concentration (and thus, high viscosity), and achieving high *M*/*A* (or *C*/*A*) Si-based anodes, are quite important and urgent.

Here we show that the goals outlined above can be simultaneously achieved by using MXene nanosheets as a new class of conductive binder to fabricate high-*M*/*A* Si/MXene anodes without any additional polymer or CB. MXenes are an emerging class of two-dimensional (2D) materials produced by selectively etching the A-group element (typically Al or Ga) from the parent MAX phase^25–27^. The as-obtained MXene can be expressed in a general formula M_*n*+1_X_*n*_T_*x*_, where M represents an early transition metal, X is C and/or N, T_*x*_ stands for various surface functionalities such as –OH, –O, and/or –F, and *n* = 1, 2, or 3^28–30^. We take advantage of the excellent mechanical properties of the nanosheets to facilitate the formation of thick electrodes while their high conductivity yields a conducting network, which can efficiently distribute charge. We demonstrate two types of MXene inks, titanium carbide (Ti_3_C_2_T_*x*_) and carbonitride (Ti_3_CNT_*x*_) as the conductive binder for producing high *C*/*A* nanoscale Si/MXene anodes. We also show graphene-wrapped Si microparticles embedded in the Ti_3_C_2_T_*x*_ network enable much higher *M*/*A* with *C*/*A* compared to other Si/conductive binder systems.

## Results and discussion

### MXene ink characterization

**Fig. 1:**
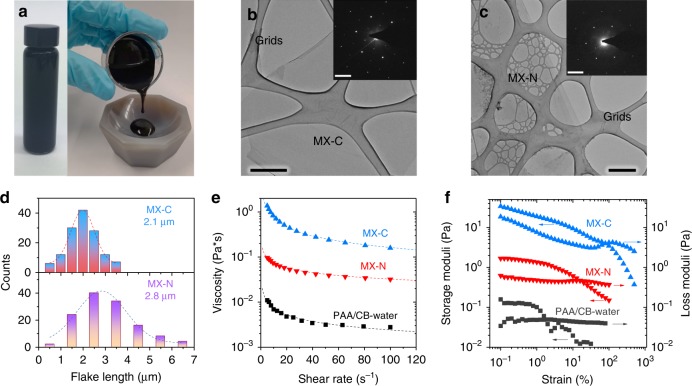
Characterization of MXene inks. **a** Optical images of the MX-C ink, showing its viscous nature. Transmission electron microscopy (TEM) images of **b** MX-C and **c** MX-N nanosheets (scale bar for **b** and **c** = 500 nm). Insets are the corresponding selected area electron diffraction patterns of the nanosheets (scale bar = 5 nm^−1^). **d** Histograms of MX-C and MX-N flake lengths obtained via TEM statistics. **e**, **f** Rheological properties of MX-C and MX-N inks with **e** viscosity plotted as a function of shear rate and **f** storage and loss moduli plotted as a function of strain. Also included is the control sample made of PAA (aqueous binder) and carbon black (CB) dispersed in water (PAA/CB-water)

We start by describing the synthesis of Ti_3_C_2_T_*x*_ (MX-C) and Ti_3_CNT_*x*_ (MX-N). After etching the MAX precursors (Supplementary Fig. 1a, b) in hydrochloric acid-lithium fluoride solution^31,32^, multilayered MXenes, with a certain degree of delamination (Supplementary Fig. 1c, d)^32^, were obtained. Upon vigorous manual shaking of the MXene/water suspension, the clay-like m-MXenes further swelled and delaminated into MX-C and MX-N flakes, forming concentrated aqueous inks. The viscous feature of the MX-C ink is shown in Fig. 1a. Both MX-C/MX-N inks are made of clean flakes with a hexagonal atomic structure (Fig. 1b, c and Supplementary Fig. 2a,b), agreeing with previous reports^12,31^. The atomic force microscopy and height profiles (Supplementary Fig. 2c) suggest these nanosheets are predominantly single-layered. These two types of inks possess a similar concentration (~25 mg mL^−1^), and the mean flake size is in the range of 2.1–2.8 µm in both cases (Fig. 1d).

As mentioned above, the CCT of Si-based anode is directly related to the viscosity of the slurry^24^. Therefore, the rheological behaviours of MXene aqueous inks, together with a reference sample made of traditional dual-component additives (PAA/carbon black, CB) dispersed in water, were evaluated. All specimens (with the same solid concentration) demonstrate non-Newtonian characteristics and shear-thinning (pseudoplastic) behaviour^33^; and the apparent viscosity (*η*, Pa·s) decreases with shear rate (*γ*, s^−1^), as demonstrated in Fig. 1e. Such behaviour can be well modelled using the Ostwald-de Wael power law:

1 $$\eta = k\gamma ^{n - 1}$$

where *k* and *n* are the consistency and shear-thinning index, respectively^34^. The empirical parameters of the studied suspensions are summarized in Supplementary Table 1. The apparent viscosity in the MX-C ink is one and two orders of magnitude higher than that of the MX-N ink and PAA/CB-water, respectively. In addition, the viscoelastic properties of the inks, in particular, storage and loss moduli, are important. These can be used to investigate regions of linear elastic deformation, yield points and sample fluidization, and give insight into the energetics of the sample network^35^. The storage and loss moduli in these viscoelastic materials were plotted as a function of strain, showing much higher storage and loss modulus in MX-C, followed by MX-N and substantially higher than the PAA/CB-water system (Fig. 1f). Such a rheological behaviour in the MXene inks should facilitate the formation of thick Si/MXene electrodes, as will be discussed below.

### Electrode fabrication and characterization

Commercial Si powders, namely, nanoscale Si (nSi, *C*_SP_ = ~3500 mAh g^−1^, size ~80 nm, Supplementary Fig. 3a) and graphene-wrapped Si (Gr-Si, *C*_SP_ = ~2000 mAh g^−1^, superstructure size ~10 µm, Supplementary Fig. 3b–d), were chosen as models for high-capacity materials.

**Fig. 2:**
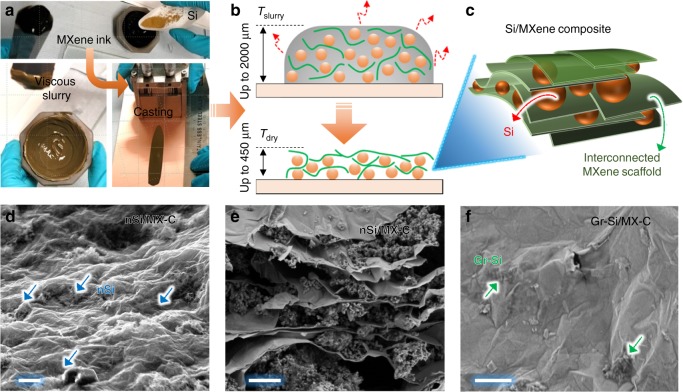
Fabrication of composite electrodes. **a** Composite electrode preparation from Si/MXene ink-based slurry. **b** The slurry drying process and **c** scheme displaying the resulting Si/MXene composite. The MXene nanosheets form a continuous scaffold and enable the formation of a thick electrode. **d** Top-view and **e** cross-sectional scanning electron microscopy (SEM) images of nSi/MX-C electrode, showing the Si nanoparticles are well wrapped by the MX-C nanosheets in a manner of sandwiching (scale bar for **d** and **e** = 1 µm). **f** Top-view SEM image of Gr-Si/MX-C electrode, indicating that the Gr-Si particles are well wrapped by the continuous MX-C network (scale bar = 1 µm)

Si powders were ground with MXene aqueous inks to give concentrated, homogenous and viscous slurry, which can be coated onto Cu foil using an industry-compatible slurry-casting technique without adding any polymeric binders or CB (Fig. 2a, Table 2 and Fig. 4). Importantly, the excellent rheological properties of MXene inks, including high viscosity, storage modulus and loss modulus enable a thick coating of the slurry (up to 650 and 2100 µm for the nSi and Gr-Si, respectively, as shown in Fig. 2b and Supplementary Table 3). In the wet slurry, the ultrathin MXene nanosheets distribute randomly and further form a continuous network while wrapping the Si particles. Upon evaporation, the capability of the nanosheet network to assimilate stress and the excellent mechanical strength of MXene nanosheets enable the formation of mechanically robust electrodes^36^, as shown in Fig. 2c. Indeed, the maximum achievable film thickness is dependent on the viscosity of the MXene ink; increasing the MXene ink concentration (or viscosity) results in thicker films (Supplementary Fig. 5). In other words, the rheological properties of the MXene ink greatly influence the electrode’s structural stability. The surface of the dried electrodes remains smooth with thickness up to ~350 µm before cracking first occurs (Supplementary Table 3), yielding a CCT much larger than the achievable thickness with traditional binders (<100 µm)^14,15,17,19,21,22^. Top-down and cross-sectional scanning electron microscopy (SEM) images with energy-dispersive X-ray mapping confirm that the nSi particulates are uniformly wrapped by the MX-C skeleton (Fig. 2d, e, Supplementary Fig. 6). No phase changes of the active materials are observed during the slurry processing, as indicated by Raman spectra (Supplementary Fig. 7) and X-ray diffraction (XRD, Supplementary Fig. 8). The nSi/MX-N exhibits a similar porous morphology as that of nSi/MX-C (Supplementary Fig. 9). On the other hand, the Gr-Si/MX-C showcases a hierarchical nano-/macro-structure, in which the Gr-Si pseudo-spherical superstructures (~10 µm) are uniformly coated with robust MX-C nanosheets (Fig. 2f and Supplementary Fig. 10). Such a morphology provides extensive free volume that allows the expansion of the nanoscale Si during the electrochemical processes, which will be discussed below.

### Electrical and mechanical characterization

**Fig. 3:**
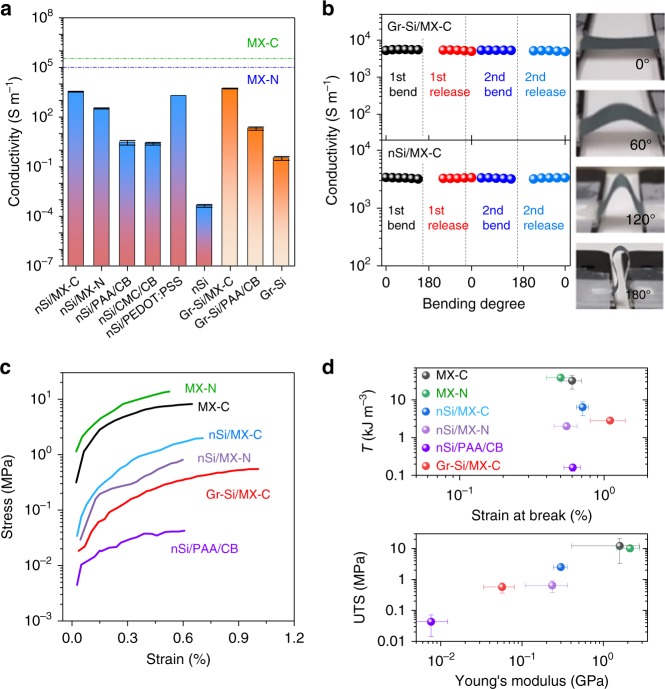
Characterization of composite electrodes. **a** Bar chart comparing the electrical conductivity comparison of various electrodes. The dashed lines are the electrical conductivity of MX-C and MX-N freestanding films. **b** Electrical conductivity change of the nSi/MX-C and Gr-Si/MX-C electrodes upon bending, as shown in the insets. The almost constant conductivity values indicate the robust nature of our Si/MXene electrodes. **c**, **d** Comparison of the mechanical properties of various electrodes. **c** Representative stress-strain curves for various electrodes. **d** Top: tensile toughness (i.e. tensile energy density required to break film) plotted as a function of strain at break (%). Bottom: tensile strength plotted as a function of Young’s modulus. The mechanical properties indicate that the MXene nanosheets confer mechanical reinforcement component, resulting in much enhanced toughness and tensile strength

To achieve high thickness and so high *C*/*A*, the Si-based anodes should possess high conductivity and mechanical toughness to ensure efficient charge transport and structural stability, respectively^37^. Previous studies revealed that the electrical conductivity issue becomes more severe in high *M*/*A* electrodes, which limit the rate capability of the electrode^38^. Here we employ MXenes to solve this issue. We performed electrical measurements on a range of Si/MXene electrodes as well as reference samples (see Supplementary Methods). Figure 3a reveals that by adding 30 wt% MXene to the Si, the conductivity has been improved roughly by ×1200 times for the nSi/MX-C (3448 S m^−1^), ×120 times for the nSi/MX-N (336 S m^−1^) and ×250 times for the Gr-Si/MX-C (5333 S m^−1^) compared to their respective traditional systems (nSi/PAA/CB and Gr-Si/PAA/CB with 70:15:15 in weight ratio). The conductivity of nSi/MX-C (70:30, *σ* = 3448 S m^−1^) was also much higher than that of nSi/CB/PAA (55:30:15, *σ* = 9.8 S m^−1^), nSi/CB/CMC (70:15:15, *σ* = 2.5 S m^−1^) and nSi/PEDOT:PSS (70:30, *σ* = 1909 S m^−1^), demonstrating the advantage of MXene in enhancing the electrical conductivity of the electrode (Fig. 3a). Moreover, the nSi/MX-C electrode conductivity scales with MX-C mass fraction (*M*_f_) and can be explained in the frame of percolation theory (Supplementary Fig. 11). Importantly, the high electrical conductivity in the nSi/MX-C and Gr-Si/MX-C electrodes can be well maintained upon repeatedly bending even in a twisted configuration (Fig. 3b), indicating the robust nature inherited from the pure MX-C film (Supplementary Fig. 12). We note this is significant as the composite electrodes (nSi/MX-C and Gr-Si/MX-C) combine high *M*/*A*, an advanced electron transport network and mechanical flexibility, holding great promise for future wearable power sources.

To further demonstrate the mechanical reinforcement provided by MXene nanosheets, the mechanical properties of Si/MXene composites (such as nSi/MX-C and Gr-Si/MX-C, nSi/MX-N), nSi/PAA/CB as well as pure MXene electrodes (MX-C, MX-N) were evaluated and compared by measuring the stress-strain curves of the corresponding vacuum-filtrated films. This is justified by the roughly similar morphologies of the samples fabricated by the two methods (Supplementary Fig. 13). The tensile toughness (area under the stress-strain curve) is of particular interest and has been improved by ×40 and ×15 in the nSi/MX-C and Gr-Si/MX-C, respectively from the traditional electrode (nSi/PAA/CB) at the equivalent composition (Si *M*_f_ = 70 wt%), as shown in Fig. 3c, d. Even though the toughness and Young’s modulus in the Si/MXene composites are lower than the pure MXene films, as expected they are higher than the traditional binder system. This allows a substantial improvement of the CCT, whose magnitude depends on the mechanical properties of the deposited materials^24^. Consequently, mechanically robust electrodes with thickness up to 350 µm for the Gr-Si/MX-C are produced. This is much higher than the achievable thickness in the traditional binder systems. In addition, it’s worth noting that the dried composite films cannot be peeled off from the Cu substrate, indicative of a strong adhesion force in the Cu/composite interface.

We note that other 2D conductive binders, in particular graphene, graphene oxide and reduced graphene oxide, typically suffer from either low ink concentration^39,40^, or poor mechanical strength of the network^41^, or un-scalable, lengthy procedures^42–44^, which greatly limit the achievable electrode mass loadings and areal capacities^44^. The superiority of concentrated MXene aqueous inks over traditional dual-component electrode additives can be attributed to the excellent electrical and mechanical properties of MXene nanosheets as well as the high ink viscosity. As a result, a simple slurry-casting technique leads to the formation of extremely thick electrodes free from any other post-treatments or complicated procedures. In the Si/MXene electrodes, the metallic conductivity of both MX-C and MX-N enables fast electron transport^45^, and thus allows reversible electrochemical reactions and high-rate performance. Furthermore, the continuous MXene network results in effective mechanical reinforcement, allowing the production of thick electrodes and potentially preserving the structural integrity of the entire electrode upon cycling. This means that the dual-functionalized MXene network should render the Si/MXene composites with good Li^+^ storage performance, as discussed below.

### Electrochemical characterization of nSi/MXene anodes

**Fig. 4:**
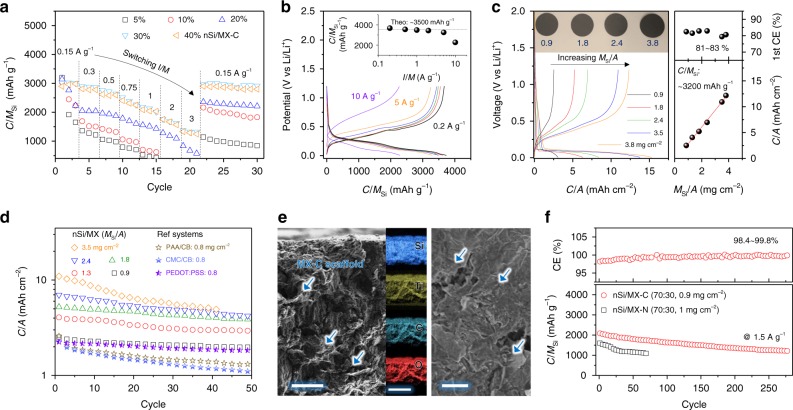
Electrochemical characterization of nSi/MXene anodes. **a** Rate performance comparison for nSi/MX-C electrodes with various MX-C mass fractions. Note that the capacity values are normalized to the mass of silicon (*C*/*M*_si_). **b** Asymmetric charge-discharge curves of the typical nSi/MX-C electrode. Inset is the as-obtained *C*/*M*_Si_ at various delithiation rates. **c** Left: first cycle charge-discharge curves at a 0.15 A g^−1^ (~1/20 C-rate) of the nSi/MX-C electrodes (MX-C *M*_f_ = 30 wt%) with the *M*_Si_/*A* ranging from 0.9 to 3.8 mg cm^−2^. Insets are the optical images of these electrodes. Right: first Coulombic efficiency (top) and areal capacity (bottom) of nSi/MX-C (MX-C *M*_f_ = 30 wt%) with various *M*_Si_/*A*. The line slope indicates the average *C*/*M*_Si_ (~3200 mAh g^−1^) achieved in various electrodes. **d** Cycling performance comparison among the electrodes with various *M*_Si_/*A*. Also included are the control samples made with PAA/carbon black (CB), CMC/CB, poly(3,4-ethylenedioxythiophene):poly(styrenesulfonic acid) (PEDOT:PSS) as the aqueous binder and conductive agent, respectively. **e** Cross-sectional (left, scale bar = 10 µm) and top-view (right, scale bar = 1 µm) scanning electron microscopy images of nSi/MX-C (MX-C *M*_f_ = 30 wt%, *M*_Si_/*A* = 2.4 mg cm^−2^) after cycling; also included is the energy-dispersive X-ray mapping, showing a uniform distribution of nSi and MX-C in the cycled electrode (scale bar = 50 µm). **f** Lifetime of nSi/MX-C and nSi/MX-N (MXene *M*_f_ = 30 wt%) electrodes at a low *M*_Si_/*A* (0.9–1 mg cm^−2^) and a high rate (1.5 A g^−1^). Also included is the Coulombic efficiency of nSi/MX-C (top)

We begin by investigating the electrochemical responses of nSi/MX-C electrodes. The d*Q*/d*V* and galvanostatic charge-discharge (GCD) profiles of nSi/MX-C electrodes (with various compositions) indicate curves that are typical for Si (Supplementary Fig. 14a and Fig. 15a–e). All electrodes show a high first Coulombic efficiency (CE) of 81–84% (Supplementary Fig. 14b). The specific capacity per nSi mass (*C*/*M*_Si_) at different current densities suggests that by adding 30 wt% MX-C conductive binder, both the rate capability (Fig. 4a) and Si electrochemical utilization are maximized, approaching the theoretical capacity (dashed line, nSi = ~3500 mAh g^−1^, Supplementary Fig. 16). Therefore, 30 wt% MXene was chosen in all Si/MXene composites. To probe the potentially achievable capacities of nSi, asymmetric charging-discharging was performed on the nSi/MX-C (*M*_f_ = 30 wt%, *M*_Si_/*A* = 0.9 mg cm^−2^); the composite was lithiated slowly (1/20 C) then delithiated at different rates (from 1/20 to 1 C). Figure 4b shows representative asymmetric GCD curves with specific capacities presented in the inset. Almost theoretical values are achieved and maintained up to 5 A g^−1^, suggesting that the high-rate response is enabled by the MX-C conductive network. Such an efficient continuous network also facilitates the production of high *M*_Si_/*A* electrodes (Fig. 4c, left panel and Supplementary Fig. 17), resulting in a *C*/*A* as high as 12.2 mAh cm^−2^ (Fig. 4c, right panel). The *C*/*A* of nSi/MX-C electrodes scales linearly with *M*_Si_/*A* over the entire thickness regime, leading to high *C*/*M*_Si_ (~3200 mAh g^−1^, dashed line) agreeing quite well with Fig. 4b. This indicates that even in the high *M*_Si_/*A* electrodes, almost theoretical capacities have been achieved due to the presence of MX-C conductive binder.

The cycling stabilities of nSi/MX-C electrodes with various *M*_Si_/*A* were measured at 0.3 A g^−1^ (Fig. 4d). While the high *M*_Si_/*A* electrode shows a relatively rapid capacity decay, reasonably stable capacities are achieved in the electrodes with low-medium range *M*_Si_/*A* (Fig. 4d and Supplementary Fig. 18). For example, the capacity retention in the *M*_Si_/*A* = 0.9 mg cm^−2^ is 84% after 50 cycles, in sharp contrast to 50% in the nSi/CB/PAA (70:15:15, *M*_Si_/*A* = 0.8 mg cm^−2^, Fig. 4d). The nSi/MX-C also outperforms other Si/conductive agent/binder electrodes, such as nSi/CB/CMC (70:15:15), nSi/PEDOT:PSS (70:30) and nSi/graphene (70:30) in cycling performance at a similar *M*_Si_/*A* (Fig. 4d and Supplementary Fig. 19). While the phase separation of Si from CB and/or PAA occurs upon repeated volume expansion/contraction. MXene nanosheets well wrap the Si particles, forming a point-to-plane contact and improving the electrochemical performance, unlike the point-to-point contact between the nSi and CB particles. The MXene skeleton conformally attaches to Si particles during repeated expanding/shrinking of the latter, which guarantees a better electron transport path, as demonstrated in Supplementary Fig. 20. Post-cycling SEM images suggest that the continuous MX-C scaffold has been preserved in the medium *M*_Si_/*A* electrode (Fig. 4e and Supplementary Fig. 21) but is disrupted in the high *M*_Si_/*A* electrode due to the large volume change (Supplementary Fig. 22). The degradation of Li metal inside the cell is also part of the reason for the relatively poor cyclability of the *M*_Si_/*A* = 3.8 mg cm^−2^ electrode (see Supplementary Fig. 22b). To further study the long-term stability of the *M*_Si_/*A* = 0.9 mg cm^−2^ electrode, a rapid charge/discharge rate was applied. The reasonable stability, coupled with high CE over 280 cycles (Fig. 4f and Supplementary Fig. 23a), can be credited to the synergistic effect between the high-capacity Si particles and the continuous MX-C network. Such a synergistic effect is not confined to MX-C; indeed any other viscous ink composed of concentrated, conductive MXene nanosheets should work as an efficient conductive binder. As a quick example, the nSi/MX-N anode delivers an initial capacity of 1602 mAh g^−1^ at 1.5 A g^−1^ and maintains 1106 mAh g^−1^ after 70 cycles (Fig. 4f and Supplementary Fig. 23b). The inferior cycling performance of nSi/MX-N anode can be possibly attributed to its lower fracture energy (toughness) compared to that of nSi/MX-C (Fig. 3d), by which the latter can assimilate the stress/tension induced by Si volume changes more efficiently. Moreover, the less thermodynamically stable and less conductive MX-N network is more prone to degradation. Despite that the cycling performance of nSi/MXene electrodes are quite comparable to other reported Si/conductive binder systems, as seen in Supplementary Table 4.

### Performance of Gr-Si/MX-C anode

**Fig. 5:**
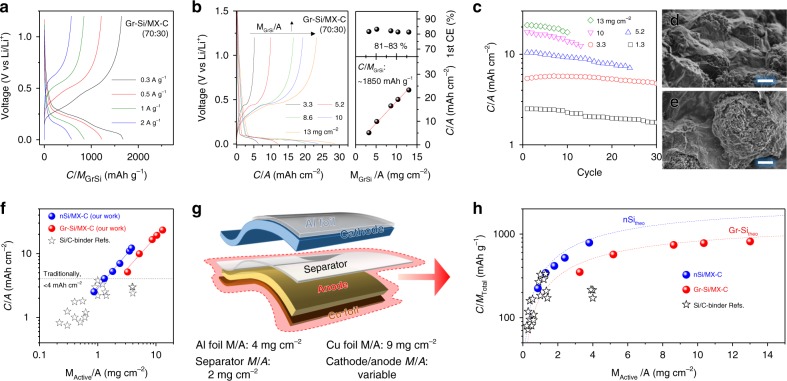
Electrochemical characterization of Gr-Si/MXene anodes and comparison to literature. **a** Typical galvanostatic charge-discharge (GCD) curves of Gr-Si/MX-C electrode (MX-C *M*_f_ = 30 wt%, *M*_GrSi_/*A* = 3.3 mg cm^−2^) at various current densities. **b** Left: GCD profiles of Gr-Si/MX-C electrodes with various *M*/*A* at 0.1 A g^−1^ (~1/20 C-rate). Right: first CE (up) and *C*/*A* (down) of Gr-Si/MX-C plotted as a function of *M*_Gr-Si_/*A*. **c** Cycling performance of Gr-Si/MX-C electrodes with various *M*_GrSi_/*A* at 0.2 A g^−1^ (~1/10 C-rate). **d**, **e** Scanning electron microscopy images of the Gr-Si/MX-C electrode (*M*_GrSi_/*A* = 3.3 mg cm^−2^) after cycling, showing that the MX-C binder tightly wrapped the Gr-Si particles and maintained the structural integrity (scale bar for **d** and **e** = 2 µm). **f** Areal capacity comparison of this work to other Si/conductive-binder (Si/C-binder) systems, showing that our Si/MX-C electrodes have exhibited both high *M*_Active_/*A* and *C*/*A* compared to the literature. **g** Scheme of components inside the cell, highlighting the importance of utilizing high *M*_Active_/*A* electrodes in reducing the contribution from the inactive components. **h** Cell-level specific capacity (*C*/*M*_Total_) on the anode side plotted as a function of *M*_Active_/*A*, and compared to the reported Si/C-binder systems. Dashed lines indicate the theoretical performance for the nSi (blue line) and Gr-Si (red line) particles

To further improve the electrode *M*/*A*, and thus *C*/*A*, we used microsized Gr-Si particles as active materials. GCD profiles (Fig. 5a and Supplementary Fig. 24a–d) of Gr-Si/MX-C electrodes with various compositions reveal typical curves for Si^46^. The first CE in these electrodes is reasonably high (81–83%, Supplementary Fig. 24e), suggesting the lithiation/delithiation processes are reversible. The rate performance suggests that 30 wt% MX-C is capable of maximizing the specific capacity at various current densities (Supplementary Fig. 25). In addition, increasing the MX-C *M*_f_ gradually not only boosts the utilization of Gr-Si to the theoretical value (*C*_SP_ = ~2000 mAh g^−1^, the dashed line in Supplementary Fig. 25c) but will also dramatically improve the mechanical properties of the electrode, according to previous findings on carbon nanotubes^37^. Thus, Gr-Si/MX-C electrodes, with *M*_GrSi_/*A* ranging from 1.3 to 13 mg cm^−2^, were fabricated at a constant composition (MX-C *M*_f_ = 30 wt%). The first GCD profiles of these Gr-Si/MX-C electrodes are shown in Fig. 5b, suggesting a high first CE (81–83%). The *C*/*A* values scale linearly with *M*_GrSi_/*A* and give a specific capacity per Gr-Si (*C*/*M*_GrSi_) as high as 1850 mAh g^−1^. The cycling performance of these electrodes is also *M*_GrSi_/*A*-dependent, being fairly stable in the *M*_GrSi_/*A* = 3.3 mg cm^−2^ electrode (*C*/*A* = ~5 mAh cm^−2^, Fig. 5c) while less stable in the *M*_GrSi_/*A* = 13 mg cm^−2^ electrode. This sharp discrepancy can be majorly attributed to the non-infinite supply of Li as well as the possible disruption of the MX-C continuous network in the thick electrode (Supplementary Fig. 26). On the other hand, intact MX-C nanosheets are well preserved and uniformly cover the Gr-Si superstructures in the *M*_GrSi_/*A* = 3.3 mg cm^−2^ electrode, as demonstrated in Fig. 5d, e.

### Comparison with published data

To demonstrate the advantage of high areal capacity Si/MXene composites for high-energy Li-ion batteries, we compared the *C*/*A* in this work to other reported Si/conductive-binder systems. The *C*/*A* of Si/MXene composites exceeds the literature results, which are generally lower than 4 mAh cm^−2^ (Fig. 5f and Supplementary Table 4)^14,17,18,20–22^. The *C*/*A* of reported Si/conductive-binder systems is typically limited by the *M*_Active_/*A* (<2 mg cm^−2^). Even with a high *M*_Active_/*A* (~4 mg cm^−2^), graphite-Si/conductive-binder electrodes display a much lower *C*/*A* than the results reported here due to the excessive graphite content in the electrodes^21,47^. The ultrahigh *C*/*A* of the Si/MXene electrodes can be attributed to the advanced electrode architecture; (1) the viscous nature of the MXene aqueous ink enables the formation of thick composite electrodes (thus high *M*_Acitve_/*A*) using a simple/scalable manufacturing process; (2) the high aspect ratio and metallic conductivity of MXene nanosheets endow the composite electrodes with mechanical robustness, high strength and excellent conductivity, facilitating fast electron transport; (3) the continuous MXene scaffold efficiently accommodates the volume change and stress induced by the Si lithiation/delithiation, and boosts the utilization of active materials. Indeed, the thickness changes are ~34% in nSi/MX-C and ~20% in Gr-Si/MX-C, suggesting 30 wt% of the MX-C nanosheet network has effectively accommodated the volume change induced by 70 wt% of Si particles (Supplementary Fig. 27).

We note that producing high *M*_Active_/*A* electrodes is of technological significance; it not only delivers a higher *C*/*A* to the electrode but also decreases the mass portion of inactive components, such as current collector (Cu foil for anode side) and separator. Consequently, the cell-level performance of the anode, which includes the mass of Cu foil (*M*_Cu-foil_/*A* = 9 mg cm^−2^), electrode and half of the separator (*M*_Separator_/*A* = 2 mg cm^−2^), can be improved significantly (Fig. 5g). Taking this into consideration, we calculated the true performance of our electrodes along with other reported Si/conductive-binder systems according to:

2 $$C/M_{{\mathrm{Total}}} = \frac{{C/A}}{{\frac{{M_{{\mathrm{Active}}}}}{A} + \frac{{M_{{\mathrm{C - binder}}}}}{A} + \frac{{M_{{\mathrm{Cu - foil}}}}}{A} + \frac{{0.5M_{{\mathrm{Separator}}}}}{A}}}$$

The *C*/*M*_Total_ was plotted versus the corresponding *M*_Active_/*A*, as shown in Fig. 5h. This comparison clearly suggests that our electrodes exhibit much higher specific capacities on the cell level, highlighting the advantages of MXene viscous inks over other conductive binders. For example, the nSi/MX-C electrode showcases a *C*/*M*_Total_ = 790 mAh g^−1^ at *M*_Si_/*A* = 3.8 mg cm^−2^, while the Gr-Si electrode demonstrates 815 mAh g^−1^ at *M*_GrSi_/*A* = 13 mg cm^−2^. Importantly, our results are quite close to the theoretical limit (dashed lines in Fig. 5h) for all samples, indicating that almost full utilization of Si active materials has been reached at our highest *M*_Acrive_/*A* electrode. Moreover, the shape of the Gr-Si/MX-C electrode is instructive; the theoretical curve (blue dashed line) nearly saturates at high *M*_GrSi_/*A* > 14 mg cm^−2^. In other words, even if we further increase the electrode thickness, the *C*/*M*_Total_ of the Gr-Si/MX-C anode will only increase marginally. This means that the electrode architecture has allowed us to reach the absolute maximum *C*/*M*_Total_ possible for the Gr-Si material used.

### Conclusion

In summary, the efficient utilization of 2D MXene nanosheets as a new class of conductive binder for high volume-change Si electrodes is of fundamental importance to the electrochemical energy storage field. The continuous network of MXene nanosheets not only provides sufficient electrical conductivity and free space for accommodating the volume change issue but also well resolves the mechanical instability of Si. Therefore, the combination of viscous MXene ink and high-capacity Si demonstrated here offers a powerful technique to construct advanced nanostructures with exceptional performance. Of equal importance is that the formation of these high-mass-loading Si/MXene electrodes can be achieved by means of a commercially compatible, slurry-casting technique, which is highly scalable and low cost, allowing for large-area production of high-performance, Si-based electrodes for advanced batteries. Considering that more than 30 MXenes are already reported, with more predicted to exist, there is certainly much room for further improving the electrochemical performance of such electrodes by tuning the electrical, mechanical and physicochemical properties of this exciting 2D MXene family.

## Methods

### MXene ink preparation

MXene viscous ink was prepared as follows: 15 mL of deionized (DI) water was added to the as-etched, multilayered MXene (Ti_3_C_2_T_*x*_ or Ti_3_CNT_*x*_), followed by vigorous shaking by hand/vortex machine for 15 min. Then the mixture was centrifuged at 3500 rpm for 30 min. The top 80% supernatant was collected and centrifuged at 5000 rpm for 30 min. After decanting the supernatant, the sediment was re-dispersed in 15 mL of DI water by vigorous shaking for 10 min, resulting in viscous MXene ink denoted as MX-C (Ti_3_C_2_T_*x*_) and MX-N (Ti_3_CNT_*x*_), respectively. A detailed description of the preparation of multilayered MXenes can be found in Supplementary Methods.

### Electrode fabrication

Electrodes were prepared via a slurry-casting method using MXene viscous aqueous ink without the addition of any other conductive additives or polymeric binder. Typically, nanosized silicon powders, nSi, (or graphene-wrapped Si, Gr-Si, Angstron Materials) were mixed with MX-C ink and ground into a uniform slurry before casting onto Cu foil using a doctor blade. After drying at ambient conditions for 2 h, electrodes were punched (12 mm in diameter) and vacuum-dried at 60 °C for 12 h to remove residual water. The MXene *M*_f_ in the electrodes (ranging from 5 to 40 wt%) was controlled by changing the mass ratio of Si powders to MXene during the slurry preparation. In addition, electrodes with different mass loadings (*M*/*A*) were obtained by changing the height of doctor blade (150–2100 µm) when casting a slurry with 30 wt% of MXene. The resultant electrodes possessed various thicknesses and thus various mass loadings and were denoted as nSi/MX-C (or Gr-Si/MX-C). We also similarly fabricated nSi/MX-N electrodes by mixing nSi powder with the MX-N viscous aqueous ink. The mass fraction of MX-N *M*_f_ in the electrodes was 30 wt%. Detailed compositions and thicknesses of the Si/MXene electrodes can be found in Supplementary Tables 2 and 3, respectively (Supplementary Methods).

### Material characterization

The rheological properties of MXene inks, as well as the reference sample, were studied on the Anton Paar MCR 301 rheometer. Morphologies and microstructure of the Si/MXene electrodes were examined by SEM, Raman spectroscopy and XRD. The electrical conductivity of the electrodes was measured using a four-point probe technique. The mechanical properties of the electrodes were measured on a Zwick Z0.5 Pro-Line Tensile Tester (100N Load Cell). A detailed description of characterization can be found in Supplementary Methods.

### Electrochemical characterization

The electrochemical performance of the Si/MXene electrodes was evaluated in the half-cell configuration (2032-type, MTI Corp.). The coin cells were assembled inside an Ar-filled glove box with Si/MXene paired with Li metal disc. A unit of 1 M lithium hexafluorophosphate (LiPF_6_) in ethylene carbonate/diethyl carbonate/fluoroethylene carbonate (3:6:1 in v/v/v, BASF) was selected as the electrolyte. GCD tests were performed within 0.005–1.2 V on a potentiostat (VMP3, BioLogic). For the post-mortem analysis, the cycled cells were carefully disassembled inside the glove box and rinsed with dimethyl carbonate. A detailed description of the electrochemical characterization can be found in Supplementary Methods.

## Supplementary Information

Supplementary Information

Source Data

## Data Availability

The data sets generated during and/or analyzed during the current study are available from the corresponding authors on reasonable request.
