# Supplementary material for: High capacity silicon anodes enabled by MXene viscous aqueous ink
Source: Nat Commun. 2019 Feb 20;10:849. doi: 10.1038/s41467-019-08383-y (PMC6382913; doi:10.1038/s41467-019-08383-y)
Supplement: Supplementary file 1 — Supplementary Information [file 41467_2019_8383_MOESM1_ESM.docx]

Supplementary Information

**High Capacity Silicon Anodes Enabled by MXene Viscous Aqueous Ink**

Chuanfang (John) Zhang *et al.*

**Supplementary Figures**

**
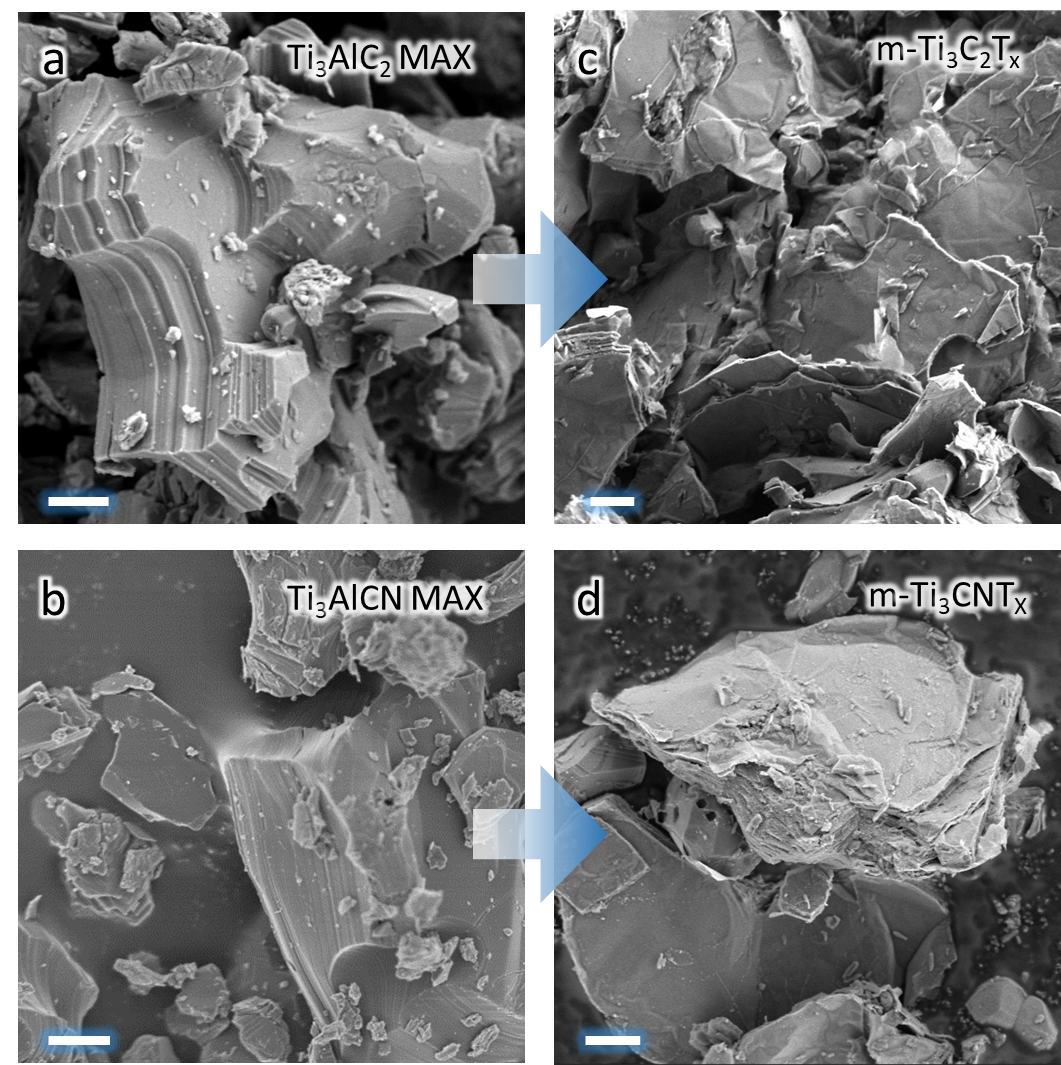
**

**Supplementary Figure 1. Morphology of MAX and MXene phases.** SEM images of (a) Ti_3_AlC_2_ and (b) Ti_3_AlCN MAX phases (scale bar for a and b = 2 µm). (c) Multi-layered (m-) Ti_3_C_2_T*_x_* and (d) m-Ti_3_CNT*_x_* prepared via the “MILD” recipe. (c–d) After washing, both m-Ti_3_C_2_T*_x_* and m-Ti_3_CNT*_x_* showcase a certain degree of delamination (scale bar for c and d = 1 µm). This is attributed to the ion exchange between the pre-intercalated Li^+^ and water molecules.

**Supplementary Figure 2. Material characterization of MXene samples.** EDX of (a) MX-C and (b) MX-N samples. (c) AFM and height profiles showing the average thickness of the samples (MX-C: ~ 1.5 nm and MX-N: ~2 nm). Both types of MXene flakes showcase a high purity (a–b) and are predominantly single-layered, as seen from the height profile in c. The thickness of MX-C flakes is ~1.5 nm while MX-N is ~2 nm.

**
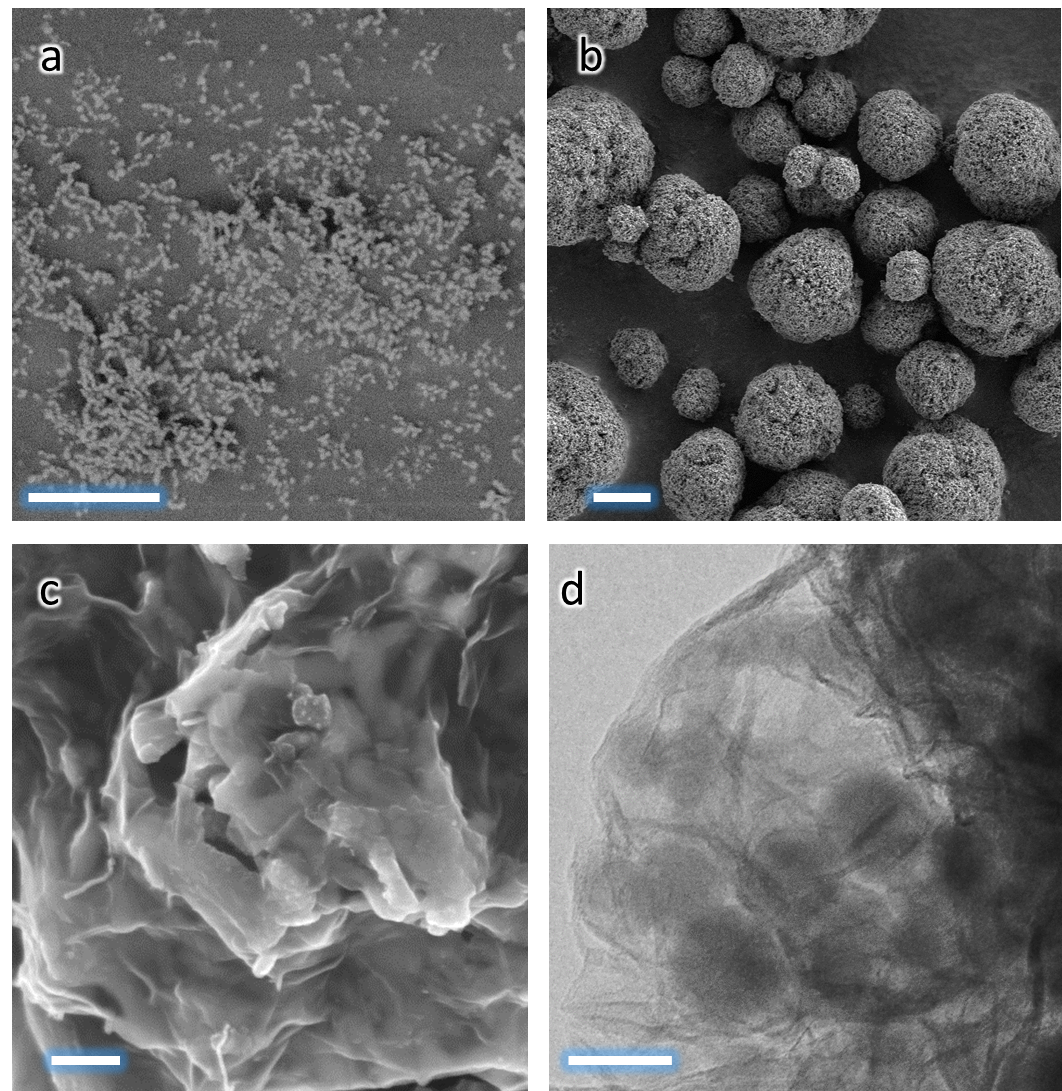
**

**Supplementary Figure 3. Morphology of bare Si materials.** SEM images of (a) bare nano-sized Si (nSi, APS = ~80 nm, C_SP_ = 3,500 mAh g^−1^, scale bar = 1 µm) and (b–c) graphene-wrapped Si powders (Gr-Si, APS = ~10 µm, C_SP_ = ~2,000 mAh g^−1^). Graphene nanosheets wrap the Si nanoparticles, forming a superstructure. Scale bar for b and c is 10 µm and 2 nm, respectively. (d) TEM image of Gr-Si, showing the presence of ultrathin graphene nanosheets (scale bar = 50 nm). It is worth noting that the Gr-Si particles are made of nanosized Si interconnected by graphene sheets, forming pseudo-spherical superstructures. The abundant voids can accommodate the large volume change of Si during lithiation/delithiation.

**
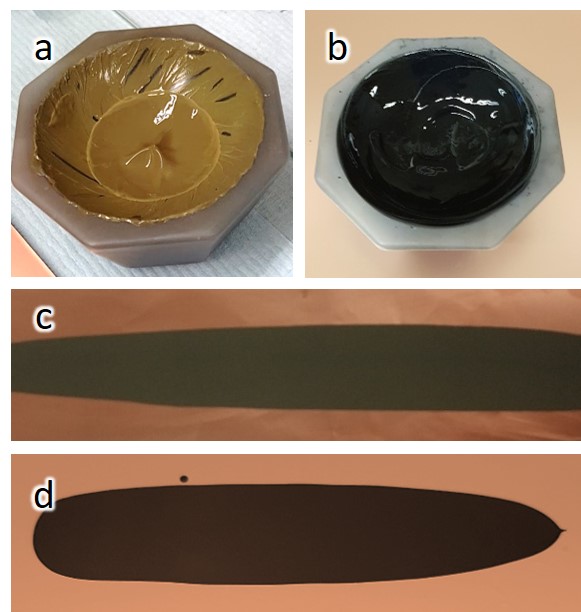
**

**Supplementary Figure 4. Preparation of Si/MXene composite anodes by slurry-casting.** Optical images of (a) nSi/MX-C and (b) Gr-Si/MX-C slurries (MXene M_f_ = 30 wt% for both cases), revealing the formation of uniform, viscous slurries that can be directly applied in the subsequent electrode casting process without adding any polymeric binders or conductive additives. Slurry-casted (c) nSi/MX-C and (d) Gr-Si/MX-C electrodes, demonstrating that MXene inks enable the production of uniform and large-area battery electrodes *via* an industry-compatible, slurry-casting route.

**Supplementary Figure 5.** **MXene ink concentration effect.** (a) Photos of the Gr-Si/MX-C electrodes prepared from different MX-C ink concentrations. (b) Slurry blade height and maximum achievable electrode M/A at various MXene ink concentrations.

**
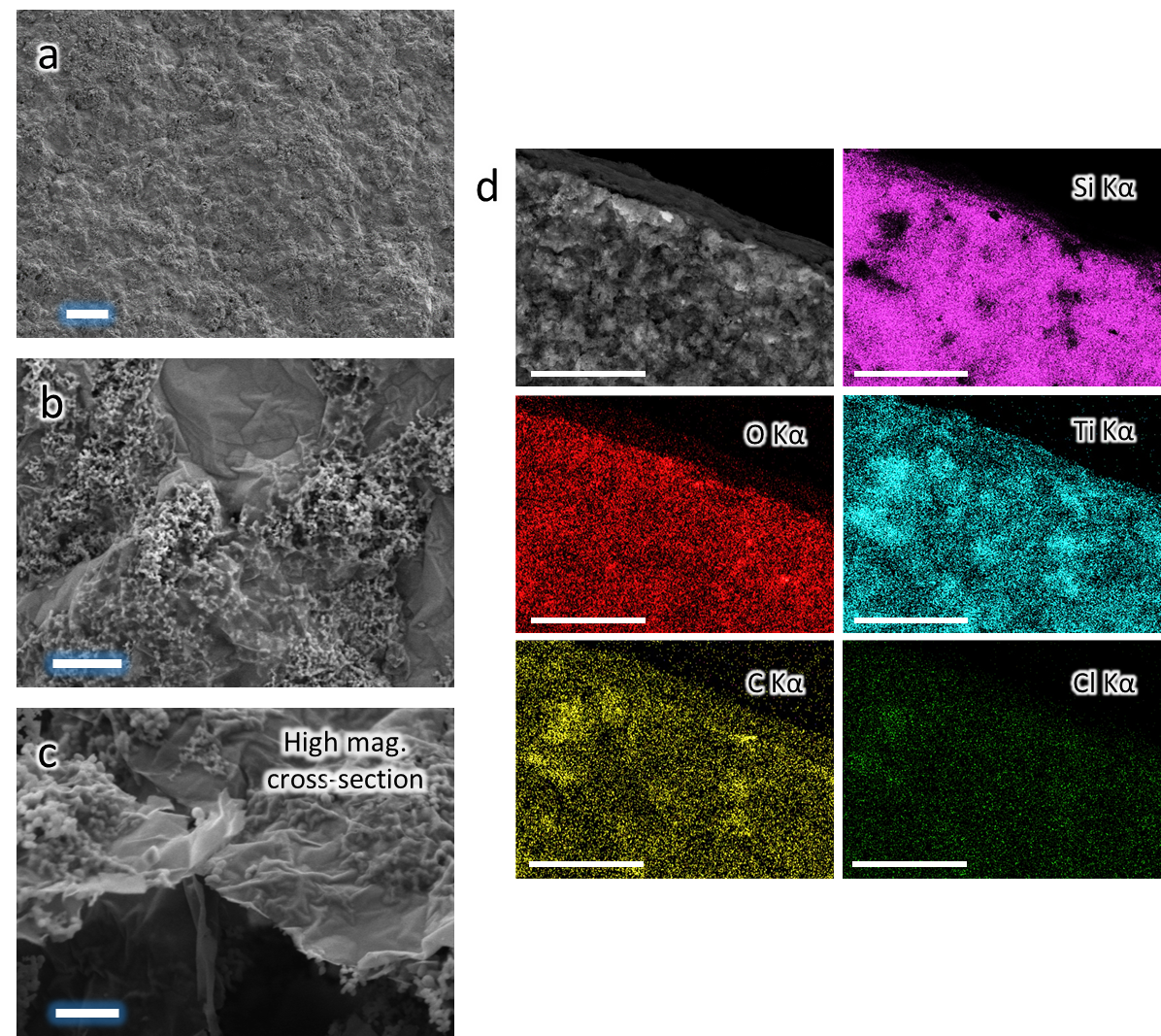
**

**Supplementary Figure 6. Morphology characterization of nSi/MX-C anodes.** (a) Low-magnification SEM image of nSi/MX-C anode, revealing the structural uniformity of the electrode (scale bar = 10 µm). (b) Top-view and (c) cross-sectional SEM images of nSi/MX-C, displaying that the nSi particles are uniformly wrapped by the interconnected MX-C network. Scale bar for b and c is 1 µm and 500 nm, respectively. (d) EDX mapping of nSi/MX-C, showing the homogeneous distribution of the elements (all scale bars = 10 µm).

**Supplementary Figure 7. Raman spectra for pure MX-C film and nSi/MX-C composite anode (MX-C M_f_ = 30 wt%).** In the composite, the characteristic peaks from MX-C well match to those for pure MX-C film, indicating no phase change during the composite-electrode fabrication process. The characteristic Si peak at ~520 cm^−1^ is evident in the composite spectrum.

**Supplementary Figure 8. XRD patterns for pure MX-C film and nSi/MX-C composite anode (MX-C M_f_ = 30 wt%).** We note that the X-ray source is Mo instead of Cu. XRD pattern of the composite corresponds well to the pure MX and Si, indicating that the crystal structures are not changed.

**
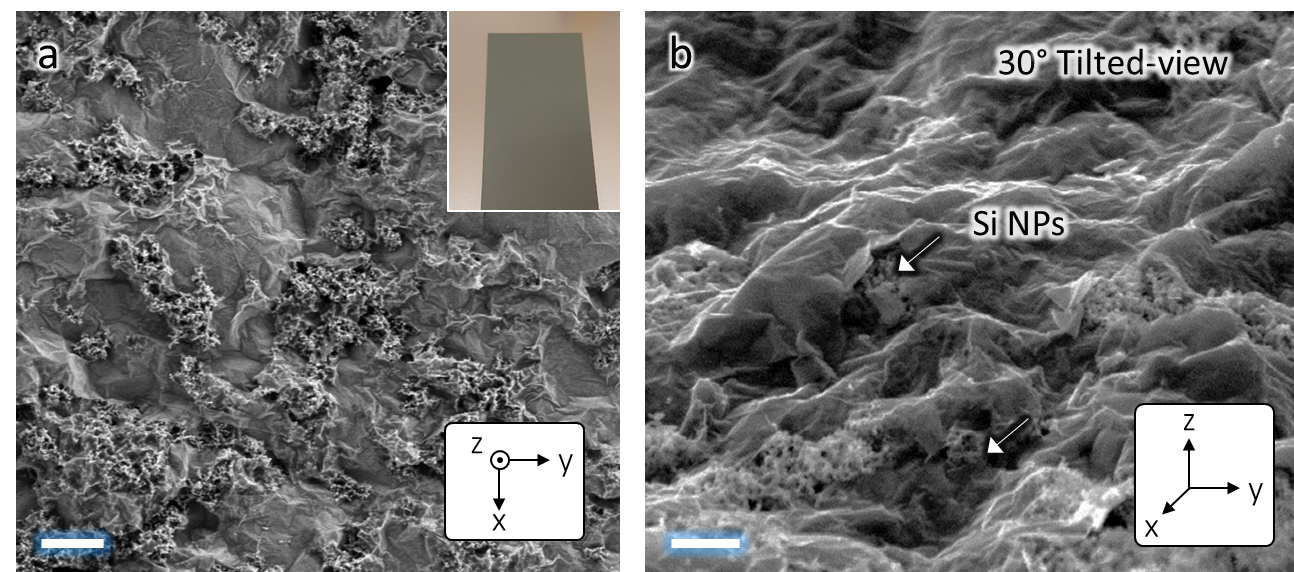
**

**Supplementary Figure 9. Morphology characterization of nSi/MX-N anodes (MX-N M_f_ = 30 wt%).** (a) Photo and top-view SEM image of slurry-casted nSi/MX-N composite anode (scale bar = 2 µm). (b) Higher-magnification SEM at a different angle, showing the morphology under the surface; the arrows indicate Si nanoparticles wrapped within MX-N nanosheets (scale bar = 1 µm).

**
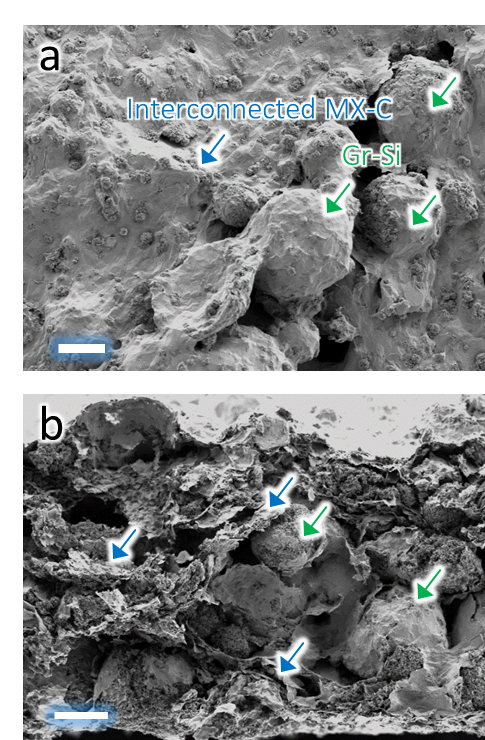
**

**Supplementary Figure 10. Morphology of Gr-Si/MX-C anodes (MX-C M_f_ = 30 wt%).** (a) Top-view and (b) cross-sectional SEM images of Gr-Si/MX-C composite anode (scale bar for a and b = 10 µm). The interconnected 2D MX-C (blue arrows) coated onto the pseudo-spherical Gr-Si particles (green arrows), ensuring rapid charge transport across the entire electrode. Moreover, the interconnected MX-C network can further accommodate the volume expansion from Gr-Si.

**Supplementary Figure 11. Electrical conductivity of nSi/MX-C as a function of MX M_f_ (5–40 wt%).** The line fits well to percolation-scaling law, $\sigma\propto{(\phi-\phi_{C,e})}^{n_{e}}$, where φ_c,e_ and n_e_ are the electrical percolation threshold and exponent, respectively.^1^ The large exponent (n_e_ = 2.2) are expected for 2D conductive networks.^2^ The percolation threshold of MX-C is 0.5 wt%, much lower than the value observed from the typically used conductive agent, carbon black (*φ*_c,e_ = 3–25 wt%).^3,4^

**Supplementary Figure 12. Electrical conductivity of pure MX-C film upon bending/releasing.** The pure MX-C film shows extremely high electrical conductivity up to ~3.6×10^5^ S m^−1^ and well maintains the conductivity upon the repeated bending/releasing test, demonstrating excellent mechanical flexibility.

**
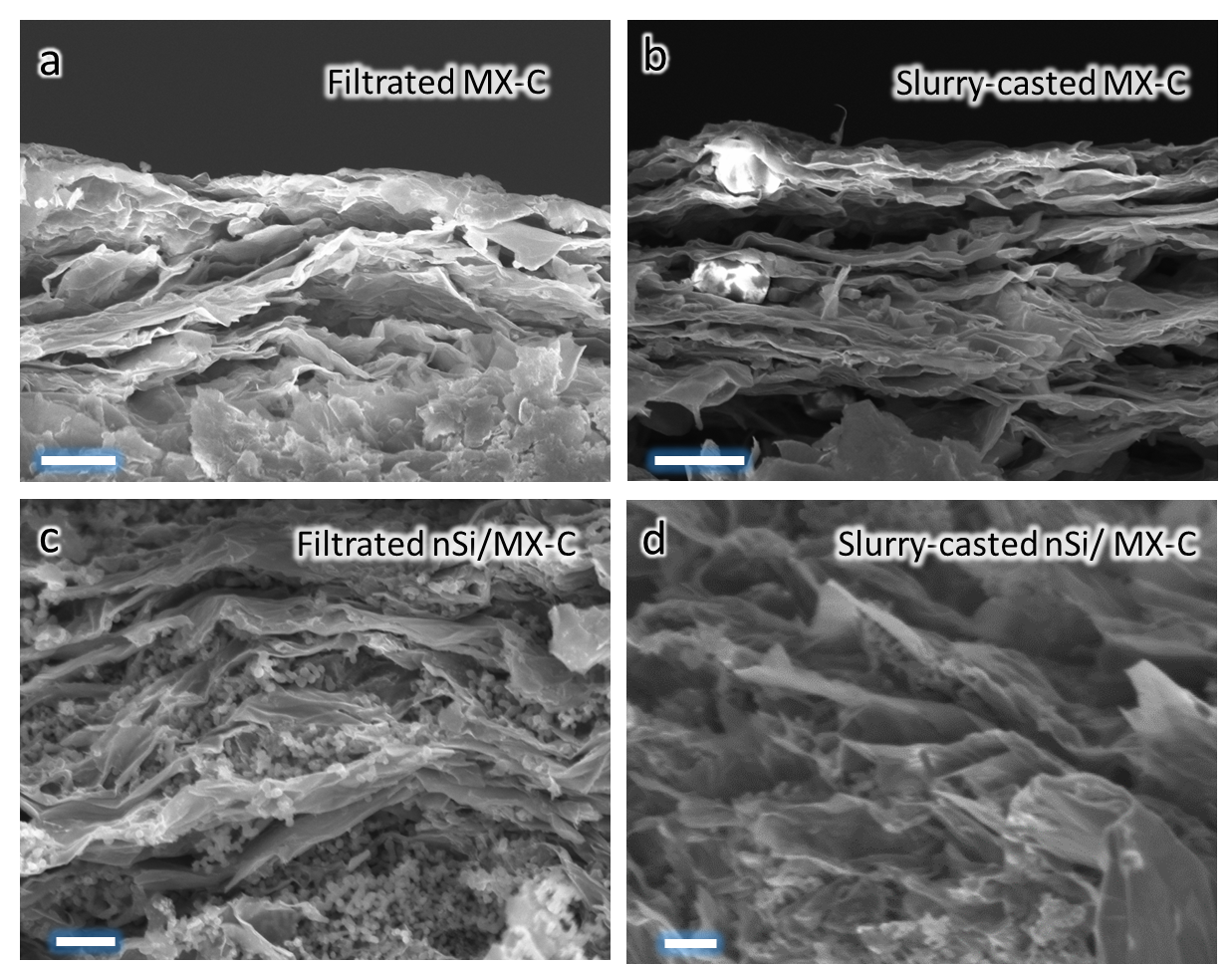
**

**Supplementary Figure 13. Morphology comparison between slurry-casting and filtration methods.** SEM images of (a) filtrated MX-C and (b) slurry-casted MX-C films, showing a layered morphology with MX-C nanosheets stacked together (scale bar for a and b = 1 µm). SEM images of (c) filtrated nSi/MX-C and (d) slurry-casted nSi/MX-C electrodes, showing a sandwiched structure with nSi particles covered by the MX-C interconnected network in both cases (scale bar for c and d = 500 nm). The SEM images of these samples confirm that, the morphology difference impacted by the electrode fabrication method is trivial. Therefore, it is reasonable and fair to estimate the mechanical performance of all the slurry-casted electrodes (such as nSi/MX-C and Gr-Si/MX-C) by measuring the strain-stress curves of the corresponding vacuum-filtrated samples, as indicated in Figure 3c–d.

**Supplementary Figure 14. Electrochemical characterization of nSi/MX-C anodes (MX-C M_f_ = 30 wt%).** (a) dQ/dV curves of nSi/MX-C (MX-C M_f_ = 30 wt%) at different cycles. (b) Bottom: 1^st^ lithiation/de-lithiation capacities of nSi/MX-C anodes as a function of MX-C M_f_. Top: 1^st^ Coulombic efficiency (CE) of nSi/MX-C anodes. From the dQ/dV curves, the nSi/MX-C electrode demonstrates typical peaks as expected for Si. A sharp cathodic peak centering at ~0.11 V which is due to the formation of solid electrolyte interphase (SEI), is found in the 1^st^ cycle and diminishes in the following cycles. Instead, cathodic peaks centering at 0.25 V and 0.1 V is found and is stable in the following cycles, which can be ascribed to the stable lithiation peaks. The first cycle Coulombic efficiency is 81–84% in the nSi/MX-C electrodes determined from the GCD profiles (Supplementary Figure 13b). It’s worth noting that Si or MXene electrodes typically exhibit a fairly low 1^st^ cycle Coulombic efficiency, for example, only ~42% and ~62% were found in the Ti_3_CNT*_x_* MXene[^2^](#_ENREF_2) and PEDOT:PSS/Si electrodes,^5,6^ respectively. The high 1^st^ Coulombic efficiency in our nSi/MXene electrodes suggest the synergistic effect between the high capacity Si particles and the MXene conductive binder, highlighting the advantage of the as-formed nanostructures.

**Supplementary Figure 15. GCD curves for nSi/MX-C anodes.** (a–e) GCD curves of nSi/MX-C anodes with different compositions (MX-C M_f_ = 5–40 wt%) at various current densities (0.15–3 A g^−1^, corresponding to 1/20–1 C rate). All GCD curves are expected for Si electrodes.

**Supplementary Figure 16.** **Si specific capacity (C/M_Si_) of nSi/MX-C anodes as a function of MX-C M_f_ (5–40 wt%).** C/M_Si_ is maximized to approach the theoretical value (dashed line, Si C_SP_ = ~3500 mAh g^−1^) at MX-C M_f_ ≥ 30 wt%, clearly suggesting the optimum MX-C M_f_ of 30 wt%.

**Supplementary Figure 17.** **Slurry-casted electrode information.** (a) M_Total_/A plotted as a function of blade height (corresponding to slurry thickness) and (b) the thickness of dried film plotted as a function of M_Total_/A. The initial concentration of materials in the Gr-Si/MX-C and nSi/MX-C slurries can be calculated as follows: (i) Considering the concentration of MX-C ink is 25 mg mL^−1^. In the composite slurries, MX-C M_f_ = 30 wt%, and Si powers M_f_ = 70 wt%; (ii) Adding Si powers to the above MX-C ink according to the above ratio gives the total concentration of slurries to be 83.3 mg mL^−1^ (= 25 mg mL^−1^ × (1 + 7/3)), assuming that the volume contribution from the Si powders can be ignored. By fitting the data in (a) gives the slope of 90 mg cm^−3^ in Gr-Si/MX-C and 81 mg cm^−3^ in nSi/MX-C, respectively, in good agreement with the initial concentration of materials in the Gr-Si/MX-C and nSi/MX-C slurry (83.3 mg cm^−3^). After drying, the thickness of the dried film dramatically shrunk. For example, the nSi/MX-C slurry shrunk from 200 µm to 35 µm, while the Gr-Si/MX-C slurry shrunk from 2100 µm to 351 µm, as shown in (b). Similarly, by fitting the data in (b) gives the density in these two Si/MXene films, being 0.65 g cc^−1^ and 0.53 g cc^−1^ in the nSi/MX-C and Gr-Si/MX-C electrodes, respectively.

**Supplementary Figure 18. Electrochemical performance of nSi/MX-C anodes with various mass loading.** Top: Si specific capacity (C/M_Si_) of nSi/MX-C electrodes with various Si mass loading (M_Si_/A) at 1^st^, 10^th^ and 25^th^ cycle. Bottom: Capacity retention of the samples from the 1^st^ cycle. As M_Si_/A increases, the capacity retention decreases; the mechanical robustness of the electrode can decrease with thickness, which renders thicker electrodes mechanically unstable and lowers the electrochemical performance.^1^

**Supplementary Figure 19. Characterization of nSi/graphene composite anodes.** (a) TEM image of the exfoliated graphene nanosheets, showing the ultrathin nature of the nanosheets (scale bar = 500 nm). The concentration of exfoliated graphene dispersion reached ~7 mg mL^−1^. Inset: photograph of graphene dispersion (b) Photos of slurry-cast nSi/graphene films. This shows that graphene nanosheets can maintain the structural stability of Si with a low mass loading (~0.4 mg cm^−2^) but fail (i.e. cracks form) when the mass loading is increased to 0.7 mg cm^−2^. (c) Electrochemical cycling of nSi/graphene electrodes tested at 1/10 C, showing a quick decay in the Si/graphene with M_Si_ = 0.7 mg cm^−2^.


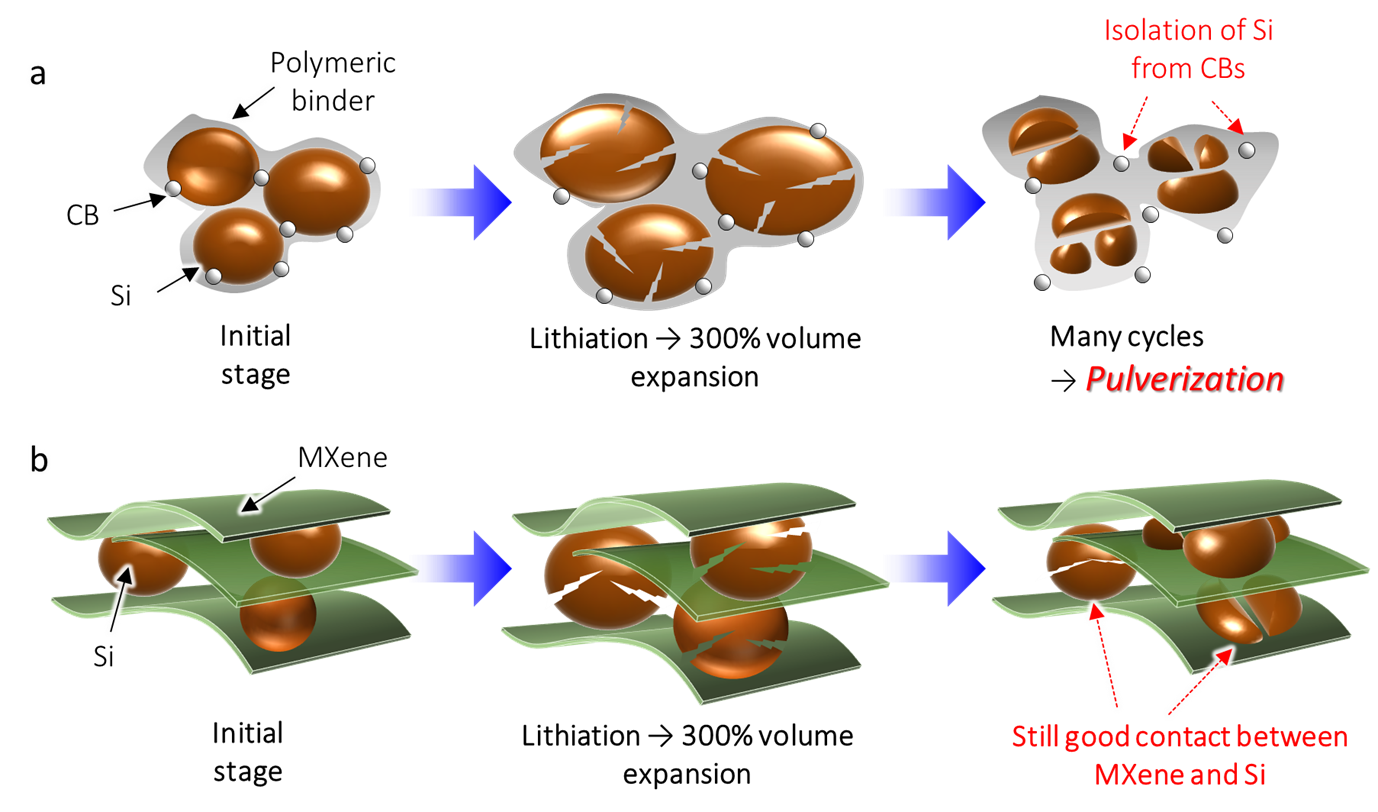


**Supplementary Figure 20.** **Comparison of electrode network change.** Schematic of the change in the electrode network upon repeated lithiation/delithiation in (a) traditional Si/conductive agent/polymeric binder and (b) Si/MXene systems.

**
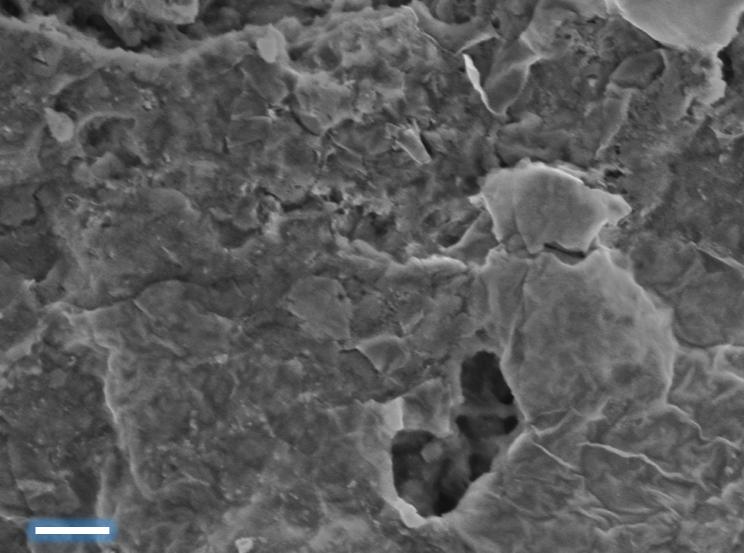
**

**Supplementary Figure 21. Higher-magnification SEM image of nSi/MX-C after cycling (M_Si_/A = 2.4 mg cm^−2^).** The MX-C nanosheets intimately covered the Si nanoparticles and maintained the structural integrity of the electrode, suggesting the capability of MX-C scaffold in accommodating the large volume change from the Si nanoparticles (scale bar = 500 nm).

**Supplementary Figure 22**. **Post-cycling analysis of nSi/MX-C with a high mass loading (M_Si_/A = 3.8 mg cm^−2^)**. (a) Cycling performance of high loading nSi/MX-C anode and (b) photo for the cycled electrodes. Li-metal counter electrode was severely degraded due to the excessive capacity of the working electrode, leading to the quick failure of the cell. (c–d) SEM images of cycled nSi/MX-C anode showing the structural degradation (scale bar for c and d = 1 µm). High-magnification in (d) displays a loose morphology due to the severe volume expansion of Si, which disrupted the MX-C interconnected network.

**Supplementary Figure 23. Electrochemical characterization of nSi/MXene anodes.** GCD curves of (a) nSi/MX-C and (b) nSi/MX-N anodes cycling at a high current density of 1.5 A g^−1^ (MXene M_f_ = 30 wt% and M_Si_ /A = ~1 mg cm^−2^ for both samples). The MX-N enabled electrodes could be cycled at a high rate with a good capacity retention.

**Supplementary Figure 24.** **Electrochemical characterization of Gr-Si/MX-C composite anodes.** (a–e) GCD curves of Gr-Si/MX-C anodes with different compositions (MX-C M_f_ = 5–40 wt%) at various current densities (0.3–2 A g^−1^, corresponding to 1/7−1 C rate). (e) Bottom: 1^st^ lithiation/de-lithiation capacities of Gr-Si/MX-C anodes as a function of MX-C wt%. Top: 1^st^ coulombic efficiency (CE) of Gr-Si/MX-C anodes. All samples show very high 1^st^ CE of 81–83% and typical GCD curves as expected for Si.

**Supplementary Figure 25. Optimization of Gr-Si/MX-C anodes by varying MX-C M_f_ (5−40 wt%).** (a) Gr-Si specific capacities (C/M_Gr-Si_) with various MX-C M_f_ measured at different current densities (0.3–2 A g^−1^, corresponding to 1/7–1 C rate). (b) C/M_Gr-Si_ plotted *versus* current densities. With increasing MX-C M_f_ (5–30 wt%), rate-capability of Gr-Si/MX-C is improved dramatically, then become saturated with further increment of MX-C M_f_ (30–40 wt%). This suggests the electrical conductivity is the limiting factor that affecting the capacities and rate response. By adding 30 wt% of MX-C viscous ink, the electrochemical response is optimized in the Gr-Si anodes. This can be verified in (c), in which the C/M_Gr-Si_ was plotted as a function of MX-C M_f_. At MX-C M_f_ ≥ 30 wt%, C/M_Gr-Si_ is close to the theoretical value (Gr-Si C_SP_ = ~2000 mAh g^−1^). Therefore in this work, 30 wt% MX-C was chosen as the optimum mass fraction for the Gr-Si particles.

**Supplementary Figure 26**. **Post-cycling SEM images of Gr-Si/MX-C with a very high mass loading (M_Gr-Si_/A = 13 mg cm^−2^).** Inset photo in (a) shows severe degradation of Li-metal after cycling (scale bar = 20 µm), similar to the high loading nSi/MX-C electrode (Supplementary Figure 14). High-magnification SEM image in (b) displays a disruption of continuous MXene scaffolds due to the huge volume expansion of the active materials in the very thick electrode (scale bar = 5 µm).

**
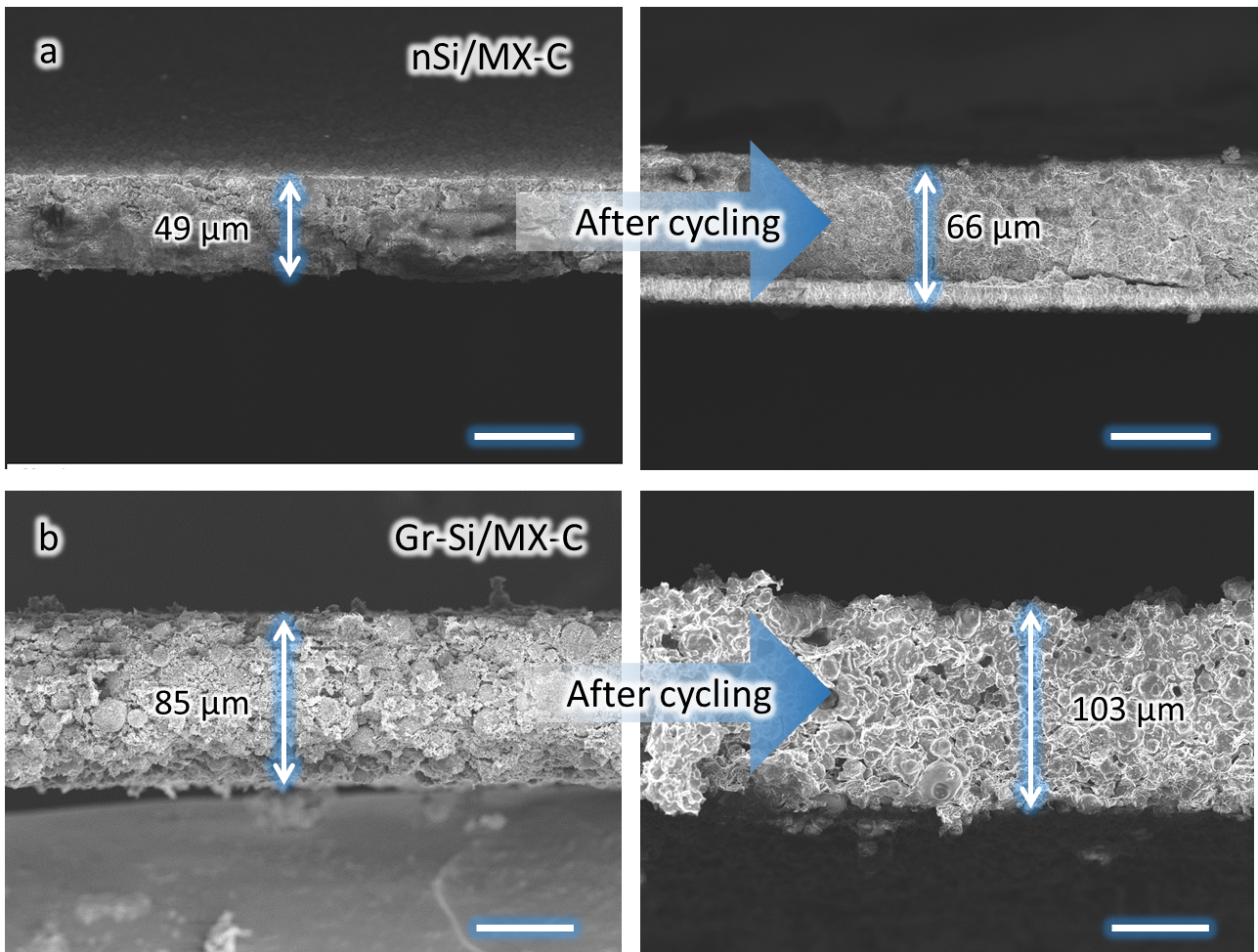
**

**Supplementary Figure 27.** **Study of the thickness change of Si/MXene anodes.** Cross-sectional SEM images of (a) nSi/MX-C and (b) Gr-Si/MX-C electrodes before (left) and after (right) cycling (all scale bar = 50 µm).

**Supplementary Tables**

**Supplementary Table 1.** Rheological parameters of MXene inks compared to PAA/CB-water

| Additives system | Solid concentration | Consistency index, k | Shear thinning index, n |
| --- | --- | --- | --- |
| MX-C ink | 25 mg mL^−1^ | 3.25 | 0.35 |
| MX-N ink | 25 mg mL^−1^ | 0.17 | 0.64 |
| PAA/CB-water | 25 mg mL^−1^ | 0.022 | 0.52 |

**Supplementary Table 2.** Si/MX-C electrodes with various formulas

| Sample conditions | Vol. of MX-C ink  (Conc. = 25 mg mL^−1^) | MX-C content | nSi (or Gr-Si) powder |
| --- | --- | --- | --- |
| 5 wt% MX-C | 0.2 mL | 5 mg | 95 mg |
| 10 wt% MX-C | 0.4 mL | 10 mg | 90 mg |
| 20 wt% MX-C | 0.8 mL | 20 mg | 80 mg |
| 30 wt% MX-C | 1.2 mL | 30 mg | 70 mg |
| 40 wt% MX-C | 1.6 mL | 40 mg | 60 mg |

**Supplementary Table 3.** Blade thickness, M/A, density and porosity of the as-fabricated Si/MXene electrodes.

| Sample | Blade thickness | Electrode thickness | Sample M_Total_/A | Sample M_Active_/A | Electrode density | Porosity |
| --- | --- | --- | --- | --- | --- | --- |
| nSi/MX-C  (MX-C  M_f_ = 30 wt%) | 150 μm | 23 μm | 1.2 mg cm^−2^ | 0.9 mg cm^−2^ | 0.52 g cc^−1^ | 80.4% |
|  | 200 μm | 35 μm | 1.9 mg cm^−2^ | 1.3 mg cm^−2^ | 0.54 g cc^−1^ | 79.6% |
|  | 300 μm | 46 μm | 2.6 mg cm^−2^ | 1.8 mg cm^−2^ | 0.57 g cc^−1^ | 78.8% |
|  | 400 μm | 53 μm | 3.4 mg cm^−2^ | 2.4 mg cm^−2^ | 0.64 g cc^−1^ | 75.9% |
|  | 600 μm | 79 μm | 5 mg cm^−2^ | 3.5 mg cm^−2^ | 0.63 g cc^−1^ | 76.2% |
|  | 650 μm | 83 μm | 5.4 mg cm^−2^ | 3.8 mg cm^−2^ | 0.65 g cc^−1^ | 75.6% |
| Gr-Si/MX-C  (MX-C  M_f_ = 30 wt%) | 600 μm | 102 μm | 4.6 mg cm^−2^ | 3.2 mg cm^−2^ | 0.45 g cc^−1^ | 81.9% |
|  | 900 μm | 150 μm | 7.4 mg cm^−2^ | 5.2 mg cm^−2^ | 0.49 g cc^−1^ | 80.2% |
|  | 1500 μm | 250 μm | 12.6 mg cm^−2^ | 8.6 mg cm^−2^ | 0.50 g cc^−1^ | 79.7% |
|  | 1800 μm | 290 μm | 14.8 mg cm^−2^ | 10.4 mg cm^−2^ | 0.51 g cc^−1^ | 79.5% |
|  | 2100 μm | 351 μm | 18.6 mg cm^−2^ | 13 mg cm^−2^ | 0.53 g cc^−1^ | 78.7% |
| Reference Si electrodes | | | | | | |
| nSi/CB/PAA (CB/PAA M_f_ = 30 wt%) | - | 21 μm | 1.1 mg cm^−2^ | ~0.8 mg cm^−2^ | 0.52 g cc^−1^ | 75% |
| nSi/CB/CMC (CB/CMC M_f_ = 30 wt%) | - | 22 μm | 1.1 mg cm^−2^ | ~0.8 mg cm^−2^ | 0.5 g cc^−1^ | 76.1% |
| nSi/PEDOT:PSS (CB/PEDOT:PSS M_f_ = 30 wt%) | - | 18 μm | 1.1 mg cm^−2^ | ~0.8 mg cm^−2^ | 0.61 g cc^−1^ | 63.3% |

*Porosity of the electrodes were calculated *via,*

$$\phi=\frac{V_{Free}}{V_{Total}}=\frac{V_{Total}-\left( V_{Si}+V_{Inactive} \right)}{V_{Total}}=\frac{V_{Total}-\left[ \left( \frac{M_{Si}}{\rho_{Si, bulk}} \right)+\left( \frac{M_{Inactive}}{\rho_{Inactive, bulk}} \right) \right]}{V_{Total}}$$

$$=\frac{V_{Total}/A-\left[ \left( \frac{M_{Si}/A}{\rho_{Si, bulk}} \right)+\left( \frac{M_{Inactive}/A}{\rho_{Inactive, bulk}} \right) \right]}{V_{Total}/A}=\frac{T_{Total}-\left[ \left( \frac{M_{Si}/A}{\rho_{Si, bulk}} \right)+\left( \frac{M_{Inactive}/A}{\rho_{Inactive, bulk}} \right) \right]}{T_{Total}}$$

where *V_Free_, V_Total_, V_Si_*, and *V_Inactive_* are electrode free volume, total electrode volume, volume occupied by Si (or Gr-Si), and inactive materials’ volume (MX or CB/binder). Then *M_Si_, M_Inactive_, ρ_Si_, ρ_Inactive_,_bulk_,* are mass and bulk density for Si and inactive materials, respectively. *T_Total_*, and *A* are electrode thickness and area.

**Supplementary Table 4.** **Literature comparison of the Si/conductive-binder systems.** Other details such as electrode composition, mass loading and specific capacity are also included. For the M/A, we sorted out the values based on the total electrode mass (Si + conductive agent/binder, M_Total_/A,) as well as on only the active material mass (M_Si_/A). Specific capacity values are presented in the same manner (both C/M_Total_ and C/M_Si_).

| Conductive-binder | Electrode composition | Mass loading (M_Total_/A and M_Si_/A) | Specific capacity  (C/M_Total_ and C/M_Si_) | Max. areal capacity (C/A) | Ref. |
| --- | --- | --- | --- | --- | --- |
| Conductive-binder based Si anodes (nano Si) | | | | | |
| **MXene ink** | **Si : MXene = 70 : 30** | **Total: 5.43** **mg cm^−2^**  **(Si: 3.8 mg cm^−2^)** | **2240 mAh g^−1^ @ 0.05 C**  **(Si: 3200 mAh g^−1^)** | **12.2 mAh cm^−2^** | **Our work** |
| RGO | Si : RGO : Carbon paper = 23 : 1 : 76 | NA | ~650 mAh g^−1^ @ 100 mA g^−1^ (Si: ~ 2800 mAh g^−1^) | NA | ^7^ |
| Self-assembled RGO | Si : RGO = 50 : 50 | Si/RGO:  0.3 ~ 0.5 mg cm^−2^ | ~1680 mAh g^−1^ @ 50 mA g^−1^ (Si: ~ 3360 mAh g^−1^) | ~0.9 mAh cm^−2^ | ^8^ |
| RGO | Si : RGO = 40 : 60 | ~1 mg cm^−2^  (Si: 0.4 mg cm^−2^) | ~1750 mAh g^−1^ @ 100 mA g^−1^  (Si: ~4300 mAh g^−1^) | ~ 1.8 mAh cm^−2^ | ^9^ |
| PFFOMB | Si : PFFOMB  = 66.6 : 33.3 | 0.45 mg cm^−2^  (~0.3 mg cm^−2^) | 1665 mAh g^−1^ @ 0.1 C  (Si: 2500 mAh g^−1^) | 0.8 mAh cm^−2^ | ^10^ |
| PEEM | Si : PEEM = 66.6 : 33.3 | 0.33 mg cm^−2^  (Si: 0.22 mg cm^−2^) | 2475 mAh g^-1^  (Si: 3750 mAh g^−1^) | 0.8 mAh cm^−2^ | ^11^ |
| PANi | Si : PANi = 75 : 25 | 0.4 ~ 0.53 mg cm^−2^  (Si 0.3~0.4 mg cm^−2^) | 1875 mAh g^−1^ @ 0.3 A g^−1^  (Si: 2500 mAh g^−1^) | 1 mAh cm^−2^ | ^12^ |
| PFP-PEG  co-polymer | Si : Co-polymer  = 90 : 10 | ~ 0.7 mg cm^−2^ (Si: 0.6 mg cm^−2^) | ~1750 mAh g^−1^ @ 1/25 C  (Si: 1575 mAh g^−1^) | ~ 1 mAh cm^−2^ | ^13^ |
| Liquid-PAN (LPAN) | Si : Graphene: LPAN = 61 : 5 : 33 | ~ 1 mg cm^−2^  (Si: 0.62 mg cm^−2^) | ~1250 mAh g^−1^ @ 500 mA g^−1^ | ~ 1.2 mAh cm^−2^ | ^14^ |
| PEFM | Si : PEFM = 66 : 33 | 0.5 mg cm^−2^  (Si: 0.3 mg cm^−2^) | 2475 mAh g^−1^ @ 1/25 C  (Si: 3750 mAh g^−1^) | 1.2 mAh cm^−2^ | ^15^ |
| PPQ | Si : PPQ = 70 : 30 | ~0.8 mg cm^−2^ (Si: 0.55 mg cm^−2^) | 2290 mAh g^−1^ @ 0.028 C  (Si: 3271 mAh g^−1^) | 1.8 mAh cm^−2^ | ^16^ |
| Biopolymer lignin | Si : Lignin = 50 : 50 | ~2.8 mg cm^−2^  (Si: 1.4 mg cm^−2^) | ~800 mAh g^−1^ @ 180 mA g^−1^  (Si: ~1600 mAh g^−1^) | ~ 2.2 mAh cm^−2^ | ^17^ |
| CNT and PEDOT:PSS | Si : CNT/PEDOT = 57 : 43 | 2 mg cm^−2^  (Si: 1 mg cm^−2^) | 1100 mAh g^−1^ @ 0.2 A g^−1^  (Si: 2180 mAh g^−1^) | 2.2 mAh cm^−2^ | ^18^ |
| PPyE | Si : PPyE  = 66.6 : 33.3 | 2 mg cm^−2^  (Si: 1.34 mg cm^−2^) | 1243 mAh g^−1^ @ 0.2 mA cm^−2^  (Si: 1866 mAh g^−1^**)** | 2.5 mAh cm^−2^ | ^19^ |
| PANI | Si : PANI  = 75 : 25 | 0.8 ~ 1.2 mg cm^−2^  (Si: 0.6 ~ 0.9 mg cm^−2^) | ~2440 mAh g^−1^ @ 200 mA g^−1^  (Si: ~3250 mAh g^−1^) | 3 mAh cm^−2^ | ^20^ |
| PEDOT:PSS | Si : PEDOT/PSS = 80 : 20 | 1.5 mg cm^−2^  (Si: 1.2 mg cm^−2^) | 2200 mAh g^−1^ @ 0.5 A g^−1^  (Si: 2750 mAh g^−1^) | 3.3 mAh cm^−2^ | ^6^ |
| PAA/PANI IPN | Si : PAA/PANI IPN = 60 : 40 | 1.6 mg cm^−2^  (Si: 1 mg cm^−2^) | 2250 mAh g^−1^ @ 0.1 C  (Si: 3750 mAh g^−1^) | 3.8 mAh cm^−2^ | ^21^ |
| PPy | Si : PPy = 66 : 33 | ~2 mg cm^−2^  (Si: 1.3 mg cm^−2^) | ~2000 mAh g^−1^ @ 0.1 C  (Si: ~3000 mAh g^−1^) | ~ 4 mAh cm^−2^ | ^22^ |
| Conductive binder-Si/carbon composite active material | | | | | |
| **MXene ink** | **Gr-Si : MXene = 70 : 30** | **Total: 18.6 mg cm^−2^**  **(Si: 13 mg cm^−2^)** | **1250 mAh g^−1^ @ 0.05 C**  **(Gr-Si : 1791 mAh g^−1^)** | **23.3 mAh cm^−2^** | **Our work** |
| PEM | Si-alloy : CB : PEM = 80 : 15 : 5 | ~4 mg cm^−2^ | ~700 mAh g^−1^ @ 1 C | 3 mAh cm^−2^ | ^23^ |
| PPy | Si : Graphite : PPy = 20 : 70 : 10 | ~3.9 mg cm^−2^ | ~800 mAh g^−1^ @ 0.1 C | ~3 mAh cm^−2^ | ^24^ |
| PPyMAA | Si : Graphite : PPyMAA = 10 : 80 : 10 | ~ 4 mg cm^−2^ | 580 mAh g^−1^ @ 0.1 C | ~ 2.4 mAh cm^−2^ | ^25^ |

**Supplementary Methods**

**Multi-layered MXene preparation**

Multi-layered MXene was prepared according to the “MILD” recipe reported by Gogotsi *et al*.^26^ Typically, 0.5 g of lithium fluoride (LiF, Sigma Aldrich, USA) were added to 10 ml, 9 M of hydrochloric acid (HCl, 37 wt%, Sigma Aldrich, USA), followed by 10 min stirring at room temperature (RT) until the formation of a clear solution. After that, 0.5 g Ti_3_AlC_2_ MAX phase (Y-Carbon Corp., Ukraine) were slowly added over the course of 20 min at room temperature (RT) under magnetic stirring, then transferred to an oil bath and reacted for 24 h at 35 ºC under continuous stirring. Once the reaction ended, the suspension was transferred to centrifuge tubes and centrifuged at 3500 rpm for 3 min. The supernatant was then decanted and 15 mL of fresh DI water was added to the suspension followed by vigorous shaking and then another round of centrifugation at 3500 rpm for 3 min. This washing process was repeated 4 times until the pH of the supernatant became ~6. The supernatant was then decanted, leaving the m-Ti_3_C_2_T*_x_* on the bottom of the centrifuge tube for making the Ti_3_C_2_T*_x_* ink.

We also prepared m-Ti_3_CNT*_x_* using a similar method.^27^ Typically, 0.5 g Ti_3_AlCN was slowly added to a solution containing 0.66 g LiF and 10 ml HCl (6 M). The reaction time was set to 16 h. All the rest of the steps were similar to the Ti_3_C_2_T*_x_* counterpart.

**Electrodes fabrication**

All electrodes were prepared *via* a slurry-casting method using the MXene viscous aqueous ink (MX-C and MX-N) as the conductive-binder with Si (nSi or Gr-Si) as the active material. No other conductive agents (such as carbon black, CB) or polymeric binder (such as polyacrylate, PAA, Sigma-Aldrich, USA) were added to the slurry, enabling an environmentally friendly, facile production of battery electrode. This is in sharp contrast to conventional electrode manufacturing, which typically involves the usage of CB or PAA. While the presence of CB and PAA unavoidably add dead volume/mass and hardly contribute to the capacities to the electrodes, the electrical conductivity of the as-fabricated electrode is also much compromised.

Here we employed the MXene viscous aqueous ink to address these issues. The MXene nanosheets provide a high electrical conductivity (~3.6 × 10^5^ S m^−1^ in MX-C and ~1 × 10^5^ S m^−1^ in MX-N).^27–29^ Moreover, the predominantly monolayer nature endows the nanosheets with high aspect ratio and flexibility, which can well accommodate the guest particles and maintain the electrode’s integrity. To highlight the advantages brought by the multifunctional MXene ink, we also prepared several reference electrodes. (1) Electrodes based on nSi, carbon black (CB, Timical Super C65, MTI Corp.) and PAA aqueous binder. The mass ratio in the reference electrodes was controlled to be 70:15:15 in the typical nSi/PAA/CB electrode. To facilitate a direct conductivity comparison, we also fabricated the nSi/CB/PAA electrode with a mass ratio of 55:30:15 and M_Si_/A of 0.8 mg cm^−2^, (2) Electrodes based on nSi, CB and carboxymethyl cellulose (CMC, MTI Corp.) with a mass ratio of 70:15:15 and M_Si_/A of 0.9 mg cm^−2^. (3) Electrodes based on nSi and PEDOT:PSS (Heraeus, Clevios TM, Corp.) with a mass ratio of 70:30 and M_Si_/A of 0.9 mg cm^−2^. (4) Electrodes based on nSi and liquid-exfoliated graphene (in N-Methyl-2-pyrrolidone, NMP, ~7 mg mL^−1^) with a mass ratio of 70:30.

To determine the optimum MXene composition, we prepared different electrodes with various MX mass fractions (M_f_), ranging from 5 to 40 wt% in both nSi/MX-C and Gr-Si/MX-C. We note that electrodes were unsuccessful (either cracking or unhomogeneous) when MX M_f_ was lower than 5 wt%. Supplementary Table 2 summarizes the formulas in these electrodes.

To show the capability of our viscous MXene ink in enabling high M/A electrodes, thus high C/A for the anode, we fabricated electrodes with various M/A (MX-C M_f_ = 30 wt%) by adjusting the height of the doctor blade. Supplementary Table 3 summarizes the height of doctor blade and the M/A of the total electrodes (M_Total_/A) and active materials (M_Active_/A).

**Material characterizations**

Delaminated MXene (MX-C and MX-N) nanosheets were observed through transmission electron microscopy (TEM, JEOL 2100, Japan). The MXene ink was first diluted and then drop-casted onto ultra-thin carbon film TEM grids. The nanosheets’ length distribution histogram was obtained by measuring sufficient numbers of flakes (*N* >100) from TEM images and taking the longest axis of the flakes as the lateral dimension. Scanning electron microscopy (SEM) images were acquired on a Zeiss Ultra Plus (Carl Zeiss, Germany) at an acceleration voltage of 2 keV. Energy-dispersive X-ray spectroscopy (EDX) and mapping were acquired with the same machine.

Atomic force microscopy (AFM) was performed on an Asylum Research MFP 3D microscope working in tapping mode with a shift of −5%. A Si tip coated with Al (Budget Sensors) and a nominal resonant frequency of 300 kHz was used. The images were acquired under ambient conditions with a scanning rate of 1 Hz and a tip resonant frequency of 300 kHz. The data were processed with Gwyddion software.

Raman spectra of MX-C and nSi/MX-C were acquired using a WITec Alpha 300 R confocal Raman microscope with an excitation wavelength of 532 nm and a spectral grating with 1800 lines/mm. By focusing the laser with a lower power (~ 300 µW) through a 20x objective, we obtained representative Raman spectra for each sample.

X-ray diffraction (XRD) patterns of MX-C and nSi/MX-C were performed on an Advance Powder X-ray diffractometer (XRD, Bruker D5000 powder diffractometer) in the Bragg-Brentano configuration operating at 40 kV using Mo-Kα radiation (*λ* = 0.71073 Å), from 2*θ* angles of 2 to 35° and with a step size of 2*θ* of 0.01 degree.

Rheological properties of the MXene inks as well as the reference samples were measured on the Anton Paar MCR 301 rheometer using a PP50, parallel plate geometry (diameter of 50 mm, gap ~1 mm). A solvent trap was utilized to minimize sample drying during the measurement. All strain sweep measurements were performed at a frequency of 1 Hz and consecutive measurements were performed, checking reproducibility, to ensure sample equilibrium had been reached. The shear rate range was selected using a standard sample of water in order to omit the lower range of measurement sensitivity and turbulence at the lower and higher end respectively.

The electrical conductivity of the samples was measured using a four-point probe method. To exclude the conductivity contribution from the Cu foil, we casted the composite slurry (made of Si materials with MXene aqueous ink) as well as the reference sample (nSi/PAA/CB) onto glass plates and dried at ambient condition. Then, four lines made of Ag paint (Agar Scientific) were brushed in parallel on the films’ surface. The sheet resistance of the films was measured using a Keithley 2400 source meter, and converted to the electrical conductivity based on the samples’ geometric information (length, width and thickness). To evaluate the mechanical properties of the Si/MXene composites, strain-stress tests were performed. Since the slurry casted films cannot be peeled off from the substrates (either Cu foil or glass), therefore, we grinded the composite slurry and then vacuum filtrated through nitrocellulose membrane (Whatman, USA). The total M/A in the MXenes as well as composite films (nSi/MX-C, nSi/MX-N and Gr-Si/MX-C) was controlled at 1–2 mg cm^−2^ (thickness ~20–30 µm) while the MXene mass fraction, M_f_, was controlled to be 30 wt.%. After filtration and naturally drying, the films were peeled off from the membrane, cut into strips and stored under vacuum for further use.

The strain-stress curves of the strips were measured on a Zwick Z0.5 Pro-Line Tensile Tester (100 N Load Cell) at a strain rate of 0.5 mm min^−1^. Each data point was obtained by averaging the results from four measurements.

To study the robustness of the Si/MXene films, the two ends of the strips were fixed to the substrates. Two lines of Ag paint were brushed onto the two ends of the strip, which was connected to the multimeter through two pieces of Ag wire. The resistance of the samples upon bending/releasing at different degrees was recorded and converted to the electrical conductivity.

**Electrochemical characterization**

Once the slurry-casted films were vacuum dried, electrodes with diameter=12 mm (geometric area: 1.13 cm^2^) were punched and weighed to determine the areal mass loading (M/A). The electrochemical properties of the Si/MXene composite electrodes were evaluated in a half-cell configuration (2032-type coin cells, MTI Corp.) which were assembled in an Ar-filled glovebox (UNIlab Pro, Mbraun). Inside the coin cell, the Si/MXene is the working electrode while the Li disc (diameter: 14 mm, MTI Corp.) is the counter and reference electrode. The separator was Celgard 2320 (Celgard, USA) and the electrolyte was 1 M lithium hexafluorophosphate (LiPF_6_) in ethylene carbonate/diethyl carbonate/fluoroethylene carbonate (EC/DEC/FEC, 3:6:1 in v/v/v, BASF).

Galvanostatic charge-discharge (GCD) tests were performed on a potentiostat (VMP3, Biologic) in a voltage range of 0.005–1.2 V. The areal capacities (C/A) of the electrodes were obtained by dividing the measured cell capacity by the geometric electrode area (1.13 cm^2^). The specific capacities of the electrodes were obtained by C/M. Here, M can be the mass of the electrode (M_total_) or the active Si material (M_Si_), resulting in the specific capacity per electrode (C/M_total_) or per active Si (C/M_Si_). To investigate the maximum accessible C/A of the electrodes, the cells were tested at a reasonably slow condition of ~1/20 C-rate (0.15 A g^−1^ for nSi anodes and 0.1 A g^−1^ for Gr-Si anodes). We also fully lithiated the electrode by discharging the cells at a very slow rate (~1/20 C-rate, 0.15 A g^−1^) then delithiated the electrode (charging the cell) at various rates, ranging from 1/20 to 1 C-rate. The cycling performance of the cells was measured at a rate of ~1/10 C (0.3 A g^−1^ for nSi anodes and 0.2 A g^−1^ for Gr-Si anodes). After cycling, the cells were disassembled, rinsed with dimethyl carbonate (DMC) several times and dried inside the glove box at room temperature before the SEM analysis.

**Supplementary References**

1. Lipatov, A. *et al.* Effect of synthesis on quality, electronic properties and environmental stability of individual monolayer Ti_3_C_2_ MXene flakes. *Adv. Electron. Mater.* **2**, 1600255 (2016).

2. Du, F. *et al.* Environmental friendly scalable production of colloidal 2D titanium carbonitride MXene with minimized nanosheets restacking for excellent cycle life lithium-ion batteries. *Electrochim. Acta* **235**, 690–699 (2017).

3. Kim, S. J. *et al.* High mass loading, binder-free MXene anodes for high areal capacity Li-ion batteries. *Electrochim. Acta* **163**, 246–251 (2015).

4. Er, D. Q., Li, J. W., Naguib, M., Gogotsi, Y. & Shenoy, V. B. Ti_3_C_2_ MXene as a high capacity electrode material for metal (Li, Na, K, Ca) ion batteries. *ACS Appl. Mater. Inter.* **6**, 11173–11179 (2014).

5. Liu, Y. P. *et al.* Electrical, mechanical, and capacity percolation leads to high-performance MoS_2_/nanotube composite lithium ion battery electrodes. *ACS Nano* **10**, 5980–5990 (2016).

6. Zhang, C. F. *et al.* Enabling flexible heterostructures for Li-Ion battery anodes based on nanotube and liquid-phase exfoliated 2D gallium chalcogenide nanosheet colloidal solutions. *Small* **13**, 1701677 (2017).

7. Shang, S. Y., Yue, Y. J. & Wang, X. E. Piezoresistive strain sensing of carbon black/silicone composites above percolation threshold. *Rev. Sci. Instrum.* **87**, 123910 (2016).

8. Koysuren, O., Yesil, S. & Bayram, G. Effect of composite preparation techniques on electrical and mechanical properties and morphology of nylon 6 based conductive polymer composites. *J. Appl. Polym. Sci.* **102**, 2520–2526 (2006).

9. Lawes, S. *et al.* Inkjet-printed silicon as high performance anodes for Li-ion batteries. *Nano Energy* **36**, 313–321 (2017).

10. Higgins, T. M. *et al.* A commercial conducting polymer as both binder and conductive additive for silicon nanoparticle-based lithium-ion battery negative electrodes. *ACS Nano* **10**, 3702–3713 (2016).

11. Tao, H. C., Xiong, L. Y., Zhu, S. C., Yang, X. L. & Zhang, L. L. Flexible binder-free reduced graphene oxide wrapped Si/carbon fibers paper anode for high-performance lithium ion batteries. *Int. J. Hydrogen Energy* **41**, 21268–21277 (2016).

12. Tang, H. *et al.* Self-assembly of Si/honeycomb reduced graphene oxide composite film as a binder-free and flexible anode for Li-ion batteries. *J. Mater. Chem. A* **2**, 5834–5840 (2014).

13. Luo, Z. P., Xiao, Q. Z., Lei, G. T., Li, Z. H. & Tang, C. J. Si nanoparticles/graphene composite membrane for high performance silicon anode in lithium ion batteries. *Carbon* **98**, 373–380 (2016).

14. Liu, G. *et al.* Polymers with tailored electronic structure for high capacity lithium battery electrodes. *Adv. Mater.* **23**, 4679 (2011).

15. Wu, M. Y. *et al.* Toward an ideal polymer binder design for high-capacity battery anodes. *J. Am. Chem. Soc.* **135**, 12048–12056 (2013).

16. Wu, H. *et al.* Stable Li-ion battery anodes by in-situ polymerization of conducting hydrogel to conformally coat silicon nanoparticles. *Nat. Commun.* **4**, 1943 (2013).

17. Yuca, N. *et al.* Highly efficient poly(fluorene phenylene) copolymer as a new class of binder for high-capacity silicon anode in lithium-ion batteries. *Int. J. Energy Res.* **42**, 1148 (2018).

18. Mi, H. W. *et al.* Three-dimensional network structure of silicon-graphene-polyaniline composites as high performance anodes for Lithium-ion batteries. *Electrochim. Acta* **190**, 1032–1040 (2016).

19. Wu, M. Y. *et al.* Manipulating the polarity of conductive polymer binders for Si-based anodes in lithium-ion batteries. *J. Mater. Chem. A* **3**, 3651–3658 (2015).

20. Kim, S. M. *et al.* Poly(phenanthrenequinone) as a conductive binder for nano-sized silicon negative electrodes. *Enery Environ. Sci.* **8**, 1538–1543 (2015).

21. Chen, T., Zhang, Q. L., Xu, J. G., Pan, J. & Cheng, Y. T. Binder-free lithium ion battery electrodes made of silicon and pyrolized lignin. *Rsc Adv.* **6**, 29308–29313 (2016).

22. Park, S. J. *et al.* Side-chain conducting and phase-separated polymeric binders for high-performance silicon anodes in lithium-ion batteries. *J. Am. Chem. Soc.* **137**, 2565–2571 (2015).

23. Yu, X. H. *et al.* Three-dimensional conductive gel network as an effective binder for high-performance Si electrodes in lithium-ion batteries. *ACS Appl. Mater. Inter.* **7**, 15961–15967 (2015).

24. Feng, M. Y., Tian, J. H., Xie, H. M., Kang, Y. L. & Shan, Z. Q. Nano-silicon/polyaniline composites with an enhanced reversible capacity as anode materials for lithium ion batteries. *J. Solid State Elect.* **19**, 1773–1782 (2015).

25. Chen, Z. *et al.* A Three-dimensionally interconnected carbon nanotube-conducting polymer hydrogel network for high-performance flexible battery electrodes. *Adv. Energy Mater.* **4**, 1400207 (2014).

26. Xun, S. D., Xiang, B., Minor, A., Battaglia, V. & Liu, G. Conductive polymer and silicon composite secondary particles for a high area-loading negative electrode. *J. Electrochem. Soc.* **160**, A1380–A1383 (2013).

27. Zhao, H. *et al.* Conductive polymer binder-enabled SiO-Sn_x_Co_y_C_z_ anode for high-energy lithium-ion batteries. *ACS Appl. Mater. Inter.* **8**, 13373–13377 (2016).

28. Zhao, H., Du, A., Ling, M., Battaglia, V. & Liu, G. Conductive polymer binder for nano-silicon/graphite composite electrode in lithium-ion batteries towards a practical application. *Electrochim. Acta* **209**, 159–162 (2016).

29. Zhao, H. *et al.* Conductive polymer binder for high-tap-density nanosilicon material for lithium-ion battery negative electrode application. *Nano Lett.* **15**, 7927–7932 (2015).
